# *N*-bipolar hypersoft sets: Enhancing decision-making algorithms

**DOI:** 10.1371/journal.pone.0296396

**Published:** 2024-01-16

**Authors:** Sagvan Y. Musa

**Affiliations:** Department of Mathematics, Faculty of Education, University of Zakho, Zakho, Iraq; Korea National University of Transportation, KOREA, DEMOCRATIC PEOPLE’S REPUBLIC OF

## Abstract

This paper introduces *N*-bipolar hypersoft (*N*-BHS) sets, a versatile extension of bipolar hypersoft (BHS) sets designed to effectively manage evaluations encompassing both binary and non-binary data, thereby exhibiting heightened versatility. The major contributions of this framework are twofold: Firstly, the *N*-BHS set introduces a parameterized representation of the universe, providing a nuanced and finite granularity in perceiving attributes, thereby distinguishing itself from conventional binary BHS sets and continuous fuzzy BHS sets. Secondly, this model signifies a new area of research aimed at overcoming limitations inherent in the *N*-bipolar soft set when handling multi-argument approximate functions. Through the strategic partitioning of attributes into distinct subattribute values using disjoint sets, the *N*-BHS set emerges as a powerful tool for effectively addressing uncertainty-related problems. In pursuit of these objectives, the paper outlines various algebraic definitions, including incomplete *N*-BHS sets, efficient *N*-BHS sets, normalized *N*-BHS sets, equivalence under normalization, *N*-BHS complements, and BHS sets derived from a threshold, exemplified through illustrative examples. Additionally, the article explores set-theoretic operations within the *N*-BHS sets framework, such as relative null/whole *N*-BHS sets, *N*-BHS subsets, and two distinct approaches to *N*-BHS extended/restricted union and intersection. Finally, it proposes and compares decision-making methodologies regarding *N*-BHS sets, including a comprehensive comparison with relevant existing models.

## 1 Introduction

The existence of uncertain data holds significant relevance across a diverse range of practical problems encompassing various fields, including physical sciences, engineering technologies, environmental sciences, and social sciences. To tackle this challenge, several mathematical theories have been formulated. These theories include fuzzy set theory [1], rough set theory [2], intuitionistic fuzzy sets [3], and probabilistic theories. These mathematical frameworks provide researchers powerful means to address diverse types of uncertainties that arise in decision-making contexts.

The concept of soft sets, introduced by Molodtsov [4], emerged as a solution to overcome limitations in previous models and has become integral in various fields, including decision-making [5–9], medical diagnosis [10], and data analysis [11]. In terms of theoretical progress, Maji et al. [12] made significant contributions by introducing fundamental algebraic operations for soft sets. Their work laid the groundwork for comprehending the mathematical structure and properties of soft sets. Building upon this foundation, Ali et al. [13] further extended the investigation by introducing additional operations and exploring their implications.

From a different perspective, there has been a rising focus on expanding the concept of soft sets to accommodate uncertain environments. Shabir and Naz [14] investigated the notion of bipolar soft sets. Maji et al. [15] proposed intuitionistic fuzzy soft sets, while Jiang et al. [16] introduced interval-valued intuitionistic fuzzy soft sets. Fatimah et al. [17] introduced *N*-soft sets as a broader concept than soft sets. Several novel hybrid models such as *N*-bipolar soft sets [18], *N*-polar soft sets [19], complex fermatean fuzzy *N*-soft sets [20], bipolar fuzzy *N*-soft sets [21], bipolar complex fuzzy N-soft set [22], bipolar complex intuitionistic fuzzy N-soft [23], probabilistic hesitant *N*-soft sets [24], fuzzy *N*-soft expert sets [25], spherical fuzzy N-soft expert sets [26], Pythagorean fuzzy N-soft expert sets [27], have been developed by researchers. These models demonstrate the effective handling of hybrid situations by *N*-soft sets. Additionally, *N*-soft sets have been utilized to establish algebraic structures [28, 29] and topological structures [30]. In-depth surveys on decision-making methods associated with hybrid soft set models were conducted by Ma et al. [31].

The concept of hypersoft (HS) sets, proposed by Smarandache [32], serves as an enhancement to handle ambiguous and uncertain data within soft set-like models. HS sets employ a multi-argument approximation mapping approach, providing increased adaptability and reliability compared to traditional soft sets. The fundamentals, characteristics, and operations of HS sets have been extensively investigated [33]. Extensions of HS sets, including rough HS sets [34], plithogenic HS sets [35], and interval-valued fuzzy HS sets [36], picture fuzzy HS sets [37], have been explored, alongside discussions on their practical applications. Asaad and Musa have utilized HS sets to examine various topological concepts [38, 39].

Based on recent surveys on hybrid HS set models, it is apparent that a significant number of researchers have been attracted to the field of HS set theory and its hybrid models. These researchers have primarily focused on two types of evaluations within these models. The first type involves binary evaluations, referred to as standard HS sets, where elements are either included or excluded from a set based on specific criteria. The second type involves evaluations using real numbers ranging from 0 to 1, known as fuzzy HS sets. However, practical problems often involve non-binary and discrete data structures. In response to this, the concept of *N*-hypersoft (*N*-HS) sets was introduced by Musa and Asaad [40] as a broader framework than HS sets. *N*-HS sets incorporate the notion of parameterized characterization of objects in the universe based on a finite number of ordered grades. In recent research, Musa and Asaad [41] introduced the concept of BHS sets, which combine HS sets and bipolarity structures [42]. BHS sets are formed by considering a collection of carefully selected parameters, as well as a set associated with parameters that have opposing meanings, referred to as the “not set of parameters.” The authors also demonstrated the application of BHS sets in decision-making problems [43]. Furthermore, in their work [44, 45], they investigated the topological structures associated with BHS sets.

The motivation of this article can be summarized as follows:

- The *N*-BHS set introduces a parameterized representation of the universe with a finite level of granularity in perceiving attributes, distinguishing it from binary BHS sets and continuous fuzzy BHS sets.
- The *N*-BHS set represents a new area of research aiming to overcome limitations of the *N*-bipolar soft set in handling multi-argument approximate functions. It partitions attributes into distinct subattribute values through disjoint sets.

The subsequent sections of the paper are structured as follows: Section 2 provides the theoretical foundation on various types of sets, including HS sets (Section 2.1), *N*-HS sets (Section 2.2), and BHS sets (Section 2.3). Section 3 introduces the extended BHS sets, namely *N*-BHS sets, along with the associated definitions. Section 4 discusses aggregate operations on *N*-BHS sets and their properties. Section 5 presents decision-making approaches applicable to *N*-BHS sets. Section 6 compares the results of algorithms using specific examples, and conducts a comparison with relevant existing models. Finally, Section 7 concludes the presentation.

## 2 Hypersoft sets, *N*-hypersoft sets, and bipolar hypersoft sets

This paper initiates by examining crucial concepts pertaining to this research, prior to delving into the subsequent discussion.

### 2.1 Hypersoft sets

In this subsection, essential definitions of HS sets crucial to this study are presented. Throughout this work, let Ω be a universal set of objects, *P*(Ω) the power set of Ω, and *E*_1_, *E*_2_, …, *E*_*n*_ be pairwise disjoint sets of parameters. Let ${\tilde{F}}_{i},{\tilde{G}}_{i}\subseteq E_{i}$ for *i* = 1, 2, …, *n*.

**Definition 1**. [4] *A soft set over* Ω *is defined as* $\left(\Delta ,{\tilde{F}}_{1}\right)$, *where* $\Delta :{\tilde{F}}_{1}\to P\left(\Omega \right)$.

**Definition 2**. [32] *An HS set over* Ω *is defined as* $\left(\Delta ,{\tilde{F}}_{1}\times {\tilde{F}}_{2}\times \ldots \times {\tilde{F}}_{n}\right)$, *where* $\Delta :{\tilde{F}}_{1}\times {\tilde{F}}_{2}\times \ldots \times {\tilde{F}}_{n}\to P\left(\Omega \right)$.

To simplify notation, let’s use *E* to represent *E*_1_ × *E*_2_ × … × *E*_*n*_, *Q*_1_ to represent ${\tilde{F}}_{1}\times {\tilde{F}}_{2}\times \ldots \times {\tilde{F}}_{n}$, and *Q*_2_ to represent ${\tilde{G}}_{1}\times {\tilde{G}}_{2}\times \ldots \times {\tilde{G}}_{n}$, where *Q*_1_, *Q*_2_ ⊆ *E*. It is important to note that each element in *Q*_1_, *Q*_2_, and *E* is an *n*-tuple element.

**Definition 3**. [33] *Consider a set of parameters Q*_1_ = {*q*_1_, *q*_2_, …, *q*_*n*_}. *The NOT set of Q*_1_, *denoted as* ¬*Q*_1_, *is defined as* ¬*Q*_1_ = {¬*q*_1_, ¬*q*_2_, …, ¬*q*_*n*_}, *where* ¬*q*_*i*_ = *not q*_*i*_ *for i* = 1, 2, …, *n*.

### 2.2 *N*-hypersoft sets

Here, significant definitions concerning *N*-HS sets are presented, as proposed by [40].

**Definition 4**. *Let* Ω *represent a universe set of objects, E denote attributes, and Q*_1_ ⊆ *E. Consider R* = {0, 1, …, *N*−1} *as a set of ordered grades, where N* ∈ {2, 3, …}. *An N-HS set over* Ω *is defined as a triple* (Δ, *Q*_1_, *N*), *where* Δ: *Q*_1_ → *P*(Ω × *R*) *and possesses the following property: for each q* ∈ *Q*_1_, *there exists a unique* (*ω*, *r*_*q*_) ∈ Ω × *R such that* (*ω*, *r*_*q*_) ∈ Δ(*q*) *or* Δ(*q*)(*ω*) = *r*_*q*_.

**Definition 5**. *Let* Ω *represent a universe set of objects, E denote attributes, and Q*_1_ ⊆ *E*. *Consider R* = {0, 1, …, *N*−1} *as a set of ordered grades, where N* ∈ {2, 3, …}. *An incomplete N-HS set over* Ω *is defined as a triple* (Δ, *Q*_1_, *N*), *where* Δ: *Q*_1_ → *P*(Ω × *R*) *and possesses the following property: for each q* ∈ *Q*_1_, *there exists at most* (*ω*, *r*_*q*_) ∈ Ω × *R such that* (*ω*, *r*_*q*_) ∈ Δ(*q*) *or* Δ(*q*)(*ω*) = *r*_*q*_.

**Definition 6**. *An N-HS set* (Δ, *Q*_1_, *N*) *is considered efficient if there exists some q* ∈ *Q*_1_ *and ω* ∈ Ω *such that* Δ(*q*)(*ω*) = *N* − 1.

**Definition 7**. *The normalized N-HS set* (Δ^*o*^, *Z*, *N*) *of an N-HS set* (Δ, *Q*_1_, *N*) *over* Ω *is defined as follows: for all q*_*j*_ ∈ *Q*_1_ *and ω*_*i*_ ∈ Ω, Δ^*o*^(*q*_*j*_)(*ω*_*i*_) = Δ(*q*_*j*_)(*ω*_*i*_) − *m*, *where m* = *min* Δ(*q*_*j*_)(*ω*_*i*_), *and Z* = {1, 2, …, *z*} *represents the index set for attributes*.

**Definition 8**. *Two N-HS sets* (Δ, *Q*_1_, *N*_1_) *and* (Δ_1_, *Q*_2_, *N*_2_) *over* Ω *are considered N-HS equal if* Δ = Δ_1_, *Q*_1_ = *Q*_2_, *and N*_1_ = *N*_2_.

**Definition 9**. *Two N-HS sets* (Δ, *Q*_1_, *N*) *and* (Δ_1_, *Q*_2_, *N*) *over* Ω *are considered equivalent if their normalized N-HS sets are equal, that is, if* $\left(\Delta^{o},Z,N\right)=\left(\Delta_{1}^{o},Z_{1},N\right)$.

**Definition 10**. *The N-HS complement of* (Δ, *Q*_1_, *N*) *is denoted and defined as* (Δ, *Q*_1_, *N*)^*c*^ = (Δ^*c*^, *Q*_1_, *N*), *where* Δ^*c*^(*q*)(*ω*) = (*N*−1)\Δ(*q*)(*ω*).

**Definition 11**. *An N-HS weak complement of* (Δ, *Q*_1_, *N*) *is defined as any N-HS set* ${\left(\Delta ,Q_{1},N\right)}^{\varpi }=\left(\Delta^{\varpi },Q_{1},N\right)$ *where* Δ^*ϖ*^ (*q*)(*ω*) ∩ Δ(*q*)(*ω*) = ∅ *for each q* ∈ *Q*_1_ and *ω* ∈ Ω.

**Definition 12**. *An N-HS top weak complement of* (Δ, *Q*_1_, *N*) *is* (Δ, *Q*_1_, *N*)^*t*^ = (Δ^*t*^, *Q*_1_, *N*) *where*

$$\Delta^{t}\left(q\right)\left(\omega \right)=\{\begin{array}{cc} N-1, & if\;\Delta (q)(\omega )<N-1\\ 0, & if\;\Delta (q)(\omega )=N-1 \end{array}$$

*for all q* ∈ *Q*_1_ *and ω* ∈ Ω.

**Definition 13**. *An N-HS bottom weak complement of* (Δ, *Q*_1_, *N*) *is* (Δ, *Q*_1_, *N*)^*b*^ = (Δ^*b*^, *Q*_1_, *N*) *where*

$$\Delta^{b}\left(q\right)\left(\omega \right)=\{\begin{array}{cc} 0, & if\;\Delta (q)(\omega )>0\\ N-1, & if\;\Delta (q)(\omega )=0 \end{array}$$

*for all q* ∈ *Q*_1_ *and* *ω* ∈ Ω.

**Definition 14**. *For a given threshold* 0 < *T* < *N* *and an N-HS set* (Δ, *Q*_1_, *N*), *the associated HS set is denoted as* (Δ^*T*^, *Q*_1_) *and is defined by the expressions*:

$$\Delta^{T}\left(q\right)\left(\omega \right)=\{\begin{array}{cc} 1, & if\;\Delta (q)(\omega )\geq T\\ 0, & otherwise \end{array}$$

*for all q* ∈ *Q*_1_ *and ω* ∈ Ω.

**Definition 15**. *An N-HS set* $\left(\tilde{\Phi },Q_{1},N\right)$ *is considered a relative null N-HS set if, for all q* ∈ *Q*_1_ *and ω* ∈ Ω, $\tilde{\Phi }\left(q\right)\left(\omega \right)=0$.

**Definition 16**. *An N-HS set* $\left(\tilde{\Omega },Q_{1},N\right)$ *is considered a relative whole N-HS set if, for all q* ∈ *Q*_1_ *and ω* ∈ Ω, $\tilde{\Omega }\left(q\right)\left(\omega \right)=N-1$.

**Definition 17**. *An N-HS set* (Δ, *Q*_1_, *N*) *is referred to as the N-HS subset of* (Δ_1_, *Q*_2_, *N*), *denoted by* (Δ, *Q*_1_, *N*) ⊑ (Δ_1_, *Q*_2_, *N*), *if Q*_1_ ⊆ *Q*_2_ *and* Δ(*q*)(*ω*) ≤ Δ_1_(*q*)(*ω*) *for all q* ∈ *Q*_1_ *and ω* ∈ Ω.

**Definition 18**. *The N-HS extended union of* (Δ, *Q*_1_, *N*_1_) *and* (Δ_1_, *Q*_2_, *N*_2_) *is denoted and defined as* (Δ, *Q*_1_, *N*_1_) ⊔_*ε*_ (Δ_1_, *Q*_2_, *N*_2_) = (Δ_2_, *Q*_1_ ∪ *Q*_2_, *max*(*N*_1_, *N*_2_)), *where for all q* ∈ *Q*_1_ ∪ *Q*_2_ *and ω* ∈ Ω:

$$\Delta_{2}\left(q\right)\left(\omega \right)=\{\begin{array}{cc} \Delta (q)(\omega ) & if\;q\in Q_{1}\backslash Q_{2}\\ \Delta_{1}\left(q\right)\left(\omega \right) & if\;q\in Q_{2}\backslash Q_{1}\\ max\{\Delta \left(q\right)\left(\omega \right),\Delta_{1}\left(q\right)\left(\omega \right)\} & if\;q\in Q_{1}\cap Q_{2} \end{array}$$

**Definition 19**. *The N-HS extended intersection of* (Δ, *Q*_1_, *N*_1_) *and* (Δ_1_, *Q*_2_, *N*_2_) *is denoted and defined as* (Δ, *Q*_1_, *N*_1_) ⊓_*ε*_ (Δ_1_, *Q*_2_, *N*_2_) = (Δ_2_, *Q*_1_ ∪ *Q*_2_, *max*(*N*_1_, *N*_2_)), *where for all q* ∈ *Q*_1_ ∪ *Q*_2_ *and ω* ∈ Ω:

$$\Delta_{2}\left(q\right)\left(\omega \right)=\{\begin{array}{cc} \Delta (q)(\omega ) & if\;q\in Q_{1}\backslash Q_{2}\\ \Delta_{1}\left(q\right)\left(\omega \right) & if\;q\in Q_{2}\backslash Q_{1}\\ min\{\Delta \left(q\right)\left(\omega \right),\Delta_{1}\left(q\right)\left(\omega \right)\} & if\;q\in Q_{1}\cap Q_{2} \end{array}$$

**Definition 20**. *The N-HS restricted union of* (Δ, *Q*_1_, *N*_1_) *and* (Δ_1_, *Q*_2_, *N*_2_) *is denoted and defined as* (Δ, *Q*_1_, *N*_1_) ⊔_ℜ_ (Δ_1_, *Q*_2_, *N*_2_) = (Δ_2_, *Q*_1_ ∩ *Q*_2_, *max*(*N*_1_, *N*_2_)), *where for all q* ∈ *Q*_1_ ∩ *Q*_2_ *and ω* ∈ Ω: Δ_2_(*q*)(*ω*) = *max*{Δ(*q*)(*ω*), Δ_1_(*q*)(*ω*)}.

**Definition 21**. *The N-HS restricted intersection of* (Δ, *Q*_1_, *N*_1_) *and* (Δ_1_, *Q*_2_, *N*_2_) *is denoted and defined as* (Δ, *Q*_1_, *N*_1_) ⊓_ℜ_ (Δ_1_, *Q*_2_, *N*_2_) = (Δ_2_, *Q*_1_ ∩ *Q*_2_, *max*(*N*_1_, *N*_2_)), *where for all q* ∈ *Q*_1_ ∩ *Q*_2_ *and ω* ∈ Ω: Δ_2_(*q*)(*ω*) = *min*{Δ(*q*)(*ω*), Δ_1_(*q*)(*ω*)}.

### 2.3 Bipolar hypersoft sets

In this subsection, essential definitions of BHS sets, crucial to this research, are provided. These definitions have been sourced from the reference cited as [41].

**Definition 22**. *A triple* (Δ, ∇, *Q*_1_) *is referred to as a BHS set over* Ω, *where* Δ *and* ∇ *are mappings given by* Δ: *Q*_1_ → *P*(Ω) *and* ∇: ¬*Q*_1_ → *P*(Ω) *with* Δ(*q*) ∩ ∇(¬*q*) = ∅ *for all q* ∈ *Q*_1_. *The BHS set* (Δ, ∇, *Q*_1_) *is represented as follows*: (Δ, ∇, *Q*_1_) = {(*q*, Δ(*q*), ∇(¬*q*)): *q* ∈ *Q*_1_ *and* Δ(*q*), ∇(¬*q*) ∈ Ω}.

**Definition 23**. *Let* (Δ, ∇, *Q*_1_) *and* (Δ_1_, ∇_1_, *Q*_2_) *be two BHS sets. Then*

1. (Δ, ∇, *Q*_1_) *is a BHS subset of* (Δ_1_, ∇_1_, *Q*_2_), *denoted by* (Δ, ∇, *Q*_1_) ⊑_*s*_ (Δ_1_, ∇_1_, *Q*_2_), *if Q*_1_ ⊆ *Q*_2_ *and* Δ(*q*) ⊆ Δ_1_(*q*), ∇_1_(¬*q*)⊆ ∇(¬*q*) *for all q* ∈ *Q*_1_.
2. (Δ, ∇, *Q*_1_) *and* (Δ_1_, ∇_1_, *Q*_2_) *are BHS equal, if* (Δ, ∇, *Q*_1_) ⊑_*s*_ (Δ_1_, ∇_1_, *Q*_2_) *and* (Δ_1_, ∇_1_, *Q*_2_) ⊑_*s*_ (Δ, ∇, *Q*_1_).
3. *If* Δ(*q*) = ∅ *and* ∇(¬*q*) = Ω *for all q* ∈ *Q*_1_, *then* (Δ, ∇, *Q*_1_) *is called a relative null BHS set and is denoted by* $\left(\tilde{\Phi },\tilde{\Omega },Q_{1}\right)$.
4. *If* Δ(*q*) = Ω *and* ∇(¬*q*) = ∅ *for all q* ∈ *Q*_1_, *then* (Δ, ∇, *Q*_1_) *is called a relative whole BHS set and is denoted by* $\left(\tilde{\Omega },\tilde{\Phi },Q_{1}\right)$.
5. *The BHS complement of* (Δ, ∇, *Q*_1_) *is a BHS set* $\left(\overset{´}{\Delta },\overset{´}{\nabla },Q_{1}\right)$ *where* $\overset{´}{\Delta }\left(q\right)=\nabla \left(\neg q\right)$ *and* $\overset{´}{\nabla }\left(\neg q\right)=\Delta \left(q\right)$ *for all q* ∈ *Q*_1_.
6. *The BHS extended union of* (Δ, ∇, *Q*_1_) *and* (Δ_1_, ∇_1_, *Q*_2_), *denoted by* (Δ, ∇, *Q*_1_) ⊔_*e*_ (Δ_1_, ∇_1_, *Q*_2_), *is a BHS set* (Δ_2_, ∇_2_, *Q*_3_), *where Q*_3_ = *Q*_1_ ∪ *Q*_2_ *and for all q* ∈ *Q*_3_:
  $$\Delta_{2}\left(q\right)=\{\begin{array}{cc} \Delta (q) & if\;q\in Q_{1}\backslash Q_{2}\\ \Delta_{1}\left(q\right) & if\;q\in Q_{2}\backslash Q_{1}\\ \Delta \left(q\right)\cup \Delta_{1}\left(q\right) & if\;q\in Q_{1}\cap Q_{2} \end{array}$$
  $$\nabla_{2}\left(\neg q\right)=\{\begin{array}{cc} \nabla (\neg q) & if\;\neg q\in \neg Q_{1}\backslash \neg Q_{2}\\ \nabla_{1}\left(\neg q\right) & if\;\neg q\in \neg Q_{2}\backslash \neg Q_{1}\\ \nabla \left(\neg q\right)\cap \nabla_{1}\left(\neg q\right) & if\;\neg q\in \neg Q_{1}\cap \neg Q_{2} \end{array}$$
7. *The BHS extended intersection of* (Δ, ∇, *Q*_1_) *and* (Δ_1_, ∇_1_, *Q*_2_), *denoted by* (Δ, ∇, *Q*_1_) ⊓_*e*_ (Δ_1_, ∇_1_, *Q*_2_), *is a BHS set* (Δ_2_, ∇_2_, *Q*_3_), *where Q*_3_ = *Q*_1_ ∪ *Q*_2_ *and for all q* ∈ *Q*_3_:
  $$\Delta_{2}\left(q\right)=\{\begin{array}{cc} \Delta (q) & if\;q\in Q_{1}\backslash Q_{2}\\ \Delta_{1}\left(q\right) & if\;q\in Q_{2}\backslash Q_{1}\\ \Delta \left(q\right)\cap \Delta_{1}\left(q\right) & if\;q\in Q_{1}\cap Q_{2} \end{array}$$
  $$\nabla_{2}\left(\neg q\right)=\{\begin{array}{cc} \nabla (\neg q) & if\;\neg q\in \neg Q_{1}\backslash \neg Q_{2}\\ \nabla_{1}\left(\neg q\right) & if\;\neg q\in \neg Q_{2}\backslash \neg Q_{1}\\ \nabla \left(\neg q\right)\cup \nabla_{1}\left(\neg q\right) & if\;\neg q\in \neg Q_{1}\cap \neg Q_{2} \end{array}$$
8. *The BHS restricted union of* (Δ, ∇, *Q*_1_) *and* (Δ_1_, ∇_1_, *Q*_2_), *denoted by* (Δ, ∇, *Q*_1_) ⊔_*r*_ (Δ_1_, ∇_1_, *Q*_2_), *is a BHS set* (Δ_2_, ∇_2_, *Q*_3_), *where Q*_3_ = *Q*_1_ ∩ *Q*_2_ *and for all q* ∈ *Q*_3_: Δ_2_(*q*) = Δ(*q*) ∪ Δ_1_(*q*) *and* ∇_2_(¬*q*) = ∇(¬*q*) ∩ ∇_1_(¬*q*).
9. *The BHS restricted intersection of* (Δ, ∇, *Q*_1_) *and* (Δ_1_, ∇_1_, *Q*_2_), *denoted by* (Δ, ∇, *Q*_1_) ⊓_*r*_ (Δ_1_, ∇_1_, *Q*_2_), *is a BHS set* (Δ_2_, ∇_2_, *Q*_3_), *where Q*_3_ = *Q*_1_ ∩ *Q*_2_ *and for all q* ∈ *Q*_3_: Δ_2_(*q*) = Δ(*q*) ∩ Δ_1_(*q*) *and* ∇_2_(¬*q*) = ∇(¬*q*) ∪ ∇_1_(¬*q*).

## 3 *N*-bipolar hypersoft sets

In this section, the novel concept of *N*-BHS sets is explored, and a variety of accompanying definitions are introduced.

**Definition 24**. *Let* Ω *represent a universe set of objects, E denote attributes, and Q*_1_ ⊆ *E*. *Consider R* = {0, 1, …, *N*−1} *as a set of ordered grades, where N* ∈ {2, 3, …}. *An N-BHS set over* Ω *is defined as a quadruple* (Δ, ∇, *Q*_1_, *N*), *where* Δ: *Q*_1_ → *P*(Ω × *R*) *and* ∇: ¬*Q*_1_ → *P*(Ω × *R*) *and possesses the following property: for each q* ∈ *Q*_1_, *there exists a unique* (*ω*, *r*_*q*_), (*ω*, *r*_¬*q*_) ∈ Ω × *R such that* (*ω*, *r*_*q*_) ∈ Δ(*q*) *and* (*ω*, *r*_¬*q*_) ∈ ∇(¬*q*), *with the condition that r*_*q*_ + *r*_¬*q*_ ≤ *N*−1.

For each attribute *q* ∈ *Q*_1_ and object *ω* ∈ Ω, there exists a unique evaluation from the assessment space *R*, denoted by *r*_*q*_ and *r*_¬*q*_, where (*ω*, *r*_*q*_), (*ω*, *r*_¬*q*_) ∈ Ω × *R* such that (*ω*, *r*_*q*_) ∈ Δ(*q*) and (*ω*, *r*_¬*q*_) ∈ ∇(¬*q*). To simplify the notation and align it with the BHS set case, it can be represented Δ(*q*)(*ω*) = *r*_*q*_ and ∇(¬*q*)(*ω*) = *r*_¬*q*_ as shorthand for (*ω*, *r*_*q*_) ∈ Δ(*q*) and (*ω*, *r*_¬*q*_) ∈ ∇(¬*q*), respectively. It will be assumed that both Ω = {*ω*_*i*_, *i* = 1, 2, …, *m*} and *Q*_1_ = {*q*_*j*_, *j* = 1, 2, …, *n*} are finite, unless stated otherwise. In this case, the *N*-BHS set can be represented in tabular form, where $\left(r_{ij}^{+},r_{ij}^{-}\right)$ denotes $\left(\omega_{i},r_{ij}^{+}\right)\in \Delta \left(q_{j}\right)$ or $\Delta \left(q_{j}\right)\left(\omega_{i}\right)=r_{ij}^{+}$, and $\left(\omega_{i},r_{ij}^{-}\right)\in \nabla \left(\neg q_{j}\right)$ or $\nabla \left(\neg q_{j}\right)\left(\omega_{i}\right)=r_{ij}^{-}$. This representation is illustrated in Table 1.

**Table 1:** Tabular form of *N*-BHS set (Δ, ∇, *Q*_1_, *N*).

(Δ, ∇, *Q*_1_, *N*)	*q*_1_	*q*_2_	…	*q*_*n*_
*ω*_1_	$\left(r_{11}^{+},r_{11}^{-}\right)$	$\left(r_{12}^{+},r_{12}^{-}\right)$	…	$\left(r_{1n}^{+},r_{1n}^{-}\right)$
*ω*_2_	$\left(r_{21}^{+},r_{21}^{-}\right)$	$\left(r_{22}^{+},r_{22}^{-}\right)$	…	$\left(r_{2n}^{+},r_{2n}^{-}\right)$
…	…	…	…	…
*ω*_*m*_	$\left(r_{m1}^{+},r_{m1}^{-}\right)$	$\left(r_{m2}^{+},r_{m2}^{-}\right)$	…	$\left(r_{mn}^{+},r_{mn}^{-}\right)$

The *N*-BHS set (Δ, ∇, *Q*_1_, *N*) can be written in this simplified form:

(Δ, ∇, *Q*_1_, *N*) = {(*q*, {*ω*, Δ(*q*)(*ω*), ∇(¬*q*)(*ω*)}): *q* ∈ *Q*_1_, *ω* ∈ Ω, and Δ(*q*)(*ω*), ∇(¬*q*)(*ω*) ∈ *R*}.

Now, let’s provide a specific example that exemplifies Definition 24, going beyond the scope of the BHS set model.

**Example 1**. *Suppose a marketing agency is looking to hire a social media strategist, and there are five candidates who have applied for the position. Let the set of candidates be denoted as* Ω = {*ω*_1_, *ω*_2_, *ω*_3_, *ω*_4_, *ω*_5_}. *The required skill set for this role includes*: $Technical\;skills={\tilde{F}}_{1}=\left\{e_{1}=content\;creation,e_{2}=campaign\;management,e_{3}=analytics\right\}$, $Advertising\;skills={\tilde{F}}_{2}=\left\{e_{4}=social\;media\;advertising,e_{5}=branding\right\}$, *and* $Copywriting\;skills={\tilde{F}}_{3}=\left\{e_{6}=good\;copywriting\right\}$, *then* $Non-technical\;skills=\neg {\tilde{F}}_{1}=\left\{\neg e_{1}=content\;deletion,\neg e_{2}=campaign\;mismanagement,\neg e_{3}=lack\;of\;analytics\right\}$, $Non-advertising\;skills=\neg {\tilde{F}}_{2}=\left\{\neg e_{4}=social\;media\;avoidance,\neg e_{5}=anti-branding\right\}$, *and* $Non-copywriting\;skills=\neg {\tilde{F}}_{3}=\left\{\neg e_{6}=poor\;copywriting\right\}$. *By combining these skills, the set is formed as* $Q_{1}={\tilde{F}}_{1}\times {\tilde{F}}_{2}\times {\tilde{F}}_{3}=\left\{q_{1}=\left(e_{1},e_{4},e_{6}\right),q_{2}=\left(e_{1},e_{5},e_{6}\right),\ldots ,q_{6}=\left(e_{3},e_{5},e_{6}\right)\right\}$, *and* $\neg Q_{1}=\neg {\tilde{F}}_{1}\times \neg {\tilde{F}}_{2}\times \neg {\tilde{F}}_{3}=\left\{\neg q_{1}=\left(\neg e_{1},\neg e_{4},\neg e_{6}\right),\neg q_{2}=\left(\neg e_{1},\neg e_{5},\neg e_{6}\right),\ldots ,\neg q_{6}=\left(\neg e_{3},\neg e_{5},\neg e_{6}\right)\right\}$. *Let R* = {0, 1, 2, 3, 4, 5} *be an ordered grades where* 0 = *Minimal Level*, 1 = *Limited Level*, 2 = *Moderate Level*, 3 = *High Level*, 4 = *Extensive Level, and* 5 = *Full Level*. *A 6-BHS set can be used to represent the assessment values of each candidate for their respective skills*.

(Δ, ∇, *Q*_1_, 6) = {(*q*_1_, {*ω*_1_, 4, 1}, {*ω*_2_, 2, 1}, {*ω*_3_, 0, 0}, {*ω*_4_, 0, 5}, {*ω*_5_, 4, 0}), (*q*_2_, {*ω*_1_, 5, 0}, {*ω*_2_, 4, 1}, {*ω*_3_, 1, 1}, {*ω*_4_, 3, 2}, {*ω*_5_, 5, 0}), (*q*_3_, {*ω*_1_, 3, 2}, {*ω*_2_, 1, 4}, {*ω*_3_, 3, 1}, {*ω*_4_, 2, 2}, {*ω*_5_, 0, 1}), (*q*_4_, {*ω*_1_, 5, 0}, {*ω*_2_, 3, 1}, {*ω*_3_, 0, 2}, {*ω*_4_, 5, 0}, {*ω*_5_, 1, 3}), (*q*_5_, {*ω*_1_, 4, 1}, {*ω*_2_, 4, 1}, {*ω*_3_, 4, 0}, {*ω*_4_, 5, 0}, {*ω*_5_, 4, 1}), (*q*_6_, {*ω*_1_, 3, 2}, {*ω*_2_, 5, 0}, {*ω*_3_, 3, 1}, {*ω*_4_, 4, 1}, {*ω*_5_, 4, 0})}.

Let’s present this information in Table 2.

**Table 2:** (Δ, ∇, *Q*_1_, 6): The candidate skills assessment.

(Δ, ∇, *Q*_1_, 6)	*q*_1_	*q*_2_	*q*_3_	*q*_4_	*q*_5_	*q*_6_
*ω*_1_	(4,1)	(5,0)	(3,2)	(5,0)	(4,1)	(3,2)
*ω*_2_	(2,1)	(4,1)	(1,4)	(3,1)	(4,1)	(5,0)
*ω*_3_	(0,0)	(1,1)	(3,1)	(0,2)	(4,0)	(3,1)
*ω*_4_	(0,5)	(3,2)	(2,2)	(5,0)	(5,0)	(4,1)
*ω*_5_	(4,0)	(5,0)	(0,1)	(1,3)	(4,1)	(4,0)

**Remark 1**. *A 2-BHS set* (Δ, ∇, *Q*_1_, 2) *can be naturally identified with a BHS set* (Δ, ∇, *Q*_1_). *Formally, the 2-BHS set is identified as* (Δ, ∇, *Q*_1_, 2), *where* Δ: *Q*_1_ → *P*(Ω × {0, 1}) *and* ∇: ¬*Q*_1_ → *P*(Ω × {0, 1}), *with the BHS set* (Δ, ∇, *Q*_1_), *where* Δ: *Q*_1_ → *P*(Ω) *and* ∇: ¬*Q*_1_ → *P*(Ω). *This identification is made by defining* Δ(*q*) = {*ω* ∈ Ω: Δ(*q*)(*ω*) = 1} *and* ∇(¬*q*) = {*ω* ∈ Ω: ∇(¬*q*) = 1}.

This can be illustrated with the following example:

**Example 2**. *Let* Ω = {*ω*_1_, *ω*_2_, *ω*_3_}. *Let* ${\tilde{F}}_{1}=\left\{e_{1},e_{2}\right\}$, ${\tilde{F}}_{2}=\left\{e_{3}\right\}$, *and* ${\tilde{F}}_{3}=\left\{e_{4}\right\}$ *then* $Q_{1}={\tilde{F}}_{1}\times {\tilde{F}}_{2}\times {\tilde{F}}_{3}=\left\{q_{1}=\left(e_{1},e_{3},e_{4}\right),q_{2}=\left(e_{2},e_{3},e_{4}\right)\right\}$. *Consider the 2-BHS set be defined as follows*:

(Δ, ∇, *Q*_1_, 2) = {(*q*_1_, {*ω*_1_, 1, 0}, {*ω*_2_, 0, 0}, {*ω*_3_, 1, 0}), (*q*_2_, {*ω*_1_, 0, 1}, {*ω*_2_, 0, 1}, {*ω*_3_, 1, 0})}.

*Now, this 2-BHS set can be identified with the BHS set* (Δ, ∇, *Q*_1_) *which is defined by*:

(Δ, ∇, *Q*_1_) = {(*q*_1_, {*ω*_1_, *ω*_3_}, ∅), (*q*_2_, {*ω*_3_}, {*ω*_1_, *ω*_2_}}.

**Remark 2**. *The grade* 0 ∈ *R*, *as mentioned in Definition 24, does not indicate incomplete or lack of information. Rather, it represents the lowest grade in the set of ordered grades*.

Based on this observation, the following definition is proposed.

**Definition 25**. *Let* Ω *represent a universe set of objects, E denote attributes, and Q*_1_ ⊆ *E*. *Consider R* = {0, 1, …, *N* − 1} *as a set of ordered grades, where N* ∈ {2, 3, …}. *An incomplete N-HS set over* Ω *is defined as a quadruple* (Δ, ∇, *Q*_1_, *N*), *where* Δ: *Q*_1_ → *P*(Ω × *R*) *and* ∇: ¬*Q*_1_ → *P*(Ω × *R*) *and possesses the following property: for each q* ∈ *Q*_1_, *there exists at most* (*ω*, *r*_*q*_), (*ω*, *r*_¬*q*_) ∈ Ω × *R such that* (*ω*, *r*_*q*_) ∈ Δ(*q*) *and* (*ω*, *r*_¬*q*_) ∈ ∇(¬*q*), *with the condition that r*_*q*_ + *r*_¬*q*_ ≤ *N* − 1.

**Remark 3**. *Indeed, any N-BHS set can be straightforwardly regarded as an* (*N* + 1)-*BHS set, or more generally, as an M-BHS set where M* > *N*.

The motivation behind this concept is that in certain scenarios, the highest grades may be present in (Δ, *Q*_1_, *N*) but not in (∇, ¬*Q*_1_, *N*), and vice versa. In some cases, the highest grades may be present in both sets. Based on this observation, the following definition is introduced.

**Definition 26**. *An N-BHS set* (Δ, ∇, *Q*_1_, *N*) *is considered positively efficient if there exists q* ∈ *Q*_1_ *and ω* ∈ Ω *such that* Δ(*q*)(*ω*) = *N* − 1 *and* ∇(¬*q*)(*ω*) = 0.

**Definition 27**. *An N-BHS set* (Δ, ∇, *Q*_1_, *N*) *is considered negatively efficient if there exists q* ∈ *Q*_1_ *and ω* ∈ Ω *such that* Δ(*q*)(*ω*) = 0 *and* ∇(¬*q*)(*ω*) = *N* − 1.

**Definition 28**. *An N-BHS set* (Δ, ∇, *Q*_1_, *N*) *is classified as totally efficient if there exists q* ∈ *Q*_1_ *and ω* ∈ Ω *such that* Δ(*q*)(*ω*) = *N* − 1 *and* ∇(¬*q*)(*ω*) = 0, *as well as* Δ(*q*)(*ω*) = 0 *and* ∇(¬*q*)(*ω*) = *N* − 1.

Next, a formalization of the concept of the bottom grade is provided.

**Definition 29**. *The normalized N-BHS set* (Δ^*o*^, ∇^*o*^, *Z*, *N*) *of an N-BHS set* (Δ, ∇, *Q*_1_, *N*) *over* Ω *is defined as follows: for all q*_*j*_ ∈ *Q*_1_ *and ω*_*i*_ ∈ Ω, Δ^*o*^(*q*_*j*_)(*ω*_*i*_) = Δ(*q*_*j*_)(*ω*_*i*_) − *m and* ∇^*o*^(¬*q*_*j*_)(*ω*_*i*_) = ∇(¬*q*_*j*_)(*ω*_*i*_) − *k where m* = *min* Δ(*q*_*j*_)(*ω*_*i*_), *k* = *min* ∇(¬*q*_*j*_)(*ω*_*i*_), *and Z* = {1, 2, …, *z*} *is the index set for attributes*.

**Definition 30**. *Two N-BHS sets* (Δ, ∇, *Q*_1_, *N*_1_) *and* (Δ_1_, ∇_1_, *Q*_2_, *N*_2_) *over* Ω *are considered N-BHS equal if* Δ = Δ_1_, ∇ = ∇_1_, *Q*_1_ = *Q*_2_, *and N*_1_ = *N*_2_.

**Definition 31**. *Two N-BHS sets* (Δ, ∇, *Q*_1_, *N*) *and* (Δ_1_, ∇_1_, *Q*_2_, *N*) *over* Ω *are considered equivalent if their normalized N-BHS sets are equal, that is*, $\left(\Delta^{o},\nabla^{o},Z_{1},N)=(\Delta_{1}^{o},\nabla_{1}^{o},Z_{2},N\right)$.

**Example 3**. *Let* Ω = {*ω*_1_, *ω*_2_, *ω*_3_}. *Let E*_1_ = {*e*_1_, *e*_2_}, *E*_2_ = {*e*_3_, *e*_4_}, *and E*_3_ = {*e*_5_} *then E* = *E*_1_ × *E*_2_ × *E*_3_ = {*q*_1_ = (*e*_1_, *e*_3_, *e*_5_), *q*_2_ = (*e*_1_, *e*_4_, *e*_5_), *q*_3_ = (*e*_2_, *e*_3_, *e*_5_), *q*_4_ = (*e*_2_, *e*_4_, *e*_5_)}. *Let Q*_1_ = {*q*_1_, *q*_2_, *q*_3_} *and Q*_2_ = {*q*_1_, *q*_2_, *q*_4_}. *Consider the two N-BHS sets* (Δ, ∇, *Q*_1_, 8) *and* (Δ_1_, ∇_1_, *Q*_2_, 8) *given in* Tables 3 *and* 4 *respectively. The same result as shown in* Table 5 *is obtained by deriving the normalized 8-BHS set from* Tables 3 *and* 4. *Therefore*, (Δ, ∇, *Q*_1_, 8) *and* (Δ_1_, ∇_1_, *Q*_2_, 8) *are equivalent*.

**Table 3:** The 8-BHS set (Δ, ∇, *Q*_1_, 8) in Example 3.

(Δ, ∇, *Q*_1_, 8)	*q*_1_	*q*_2_	*q*_3_
*ω*_1_	(1,6)	(3,4)	(3,4)
*ω*_2_	(2,5)	(1,6)	(3,4)
*ω*_3_	(3,4)	(2,4)	(1,5)

**Table 4:** The 8-BHS set (Δ_1_, ∇_1_, *Q*_2_, 8) in Example 3.

(Δ_1_, ∇_1_, *Q*_2_, 8)	*q*_1_	*q*_2_	*q*_4_
*ω*_1_	(4,3)	(6,1)	(6,1)
*ω*_2_	(5,2)	(4,3)	(6,1)
*ω*_3_	(6,1)	(5,1)	(4,2)

**Table 5:** The equivalence of (Δ, ∇, *Q*_1_, 8) and (Δ_1_, ∇_1_, *Q*_2_, 8) under normalization in Example 3.

$\left(\Delta^{o},\nabla^{o},Z_{1},8)=(\Delta_{1}^{o},\nabla_{1}^{o},Z_{2},8\right)$	1	2	3
*ω*_1_	(0,2)	(2,0)	(2,0)
*ω*_2_	(1,1)	(0,2)	(2,0)
*ω*_3_	(2,0)	(1,0)	(0,1)

**Definition 32**. *An N-BHS complement of* (Δ, ∇, *Q*_1_, *N*) *is denoted and defined as* ${\left(\Delta ,\nabla ,Q_{1},N\right)}^{\tilde{c}}=\left(\Delta^{\tilde{c}},\nabla^{\tilde{c}},Q_{1},N\right)$, *where* $\Delta^{\tilde{c}}\left(q\right)\left(\omega \right)=\nabla (\neg q)(\omega )$ *and* $\nabla^{\tilde{c}}\left(\neg q\right)\left(\omega \right)=\Delta (q)(\omega )$ *for all q* ∈ *Q*_1_ *and ω* ∈ Ω.

**Definition 33**. *An N-BHS weak complement of* (Δ, ∇, *Q*_1_, *N*) *is any N-BHS set* ${\left(\Delta ,\nabla ,Q_{1},N\right)}^{\tilde{\varpi }}=\left(\Delta^{\tilde{\varpi }},\nabla^{\tilde{\varpi }},Q_{1},N\right)$, *where* $\Delta^{\tilde{\varpi }}\left(q\right)\left(\omega \right)\cap \Delta (q)(\omega )=\emptyset$ *and* $\nabla^{\tilde{\varpi }}\left(\neg q\right)\left(\omega \right)\cap \nabla (\neg q)(\omega )=\emptyset$, *for each q* ∈ *Q*_1_ *and ω* ∈ Ω.

**Definition 34**. *An N-BHS top weak complement of* (Δ, ∇, *Q*_1_, *N*) *is* ${\left(\Delta ,\nabla ,Q_{1},N\right)}^{\tilde{t}}=\left(\Delta^{\tilde{t}},\nabla^{\tilde{t}},Q_{1},N\right)$, *where*

$$\Delta^{\tilde{t}}\left(q\right)\left(\omega \right)=\{\begin{array}{cc} N-1, & if\;\Delta (q)(\omega )<N-1\\ 0, & if\;\Delta (q)(\omega )=N-1 \end{array}$$

*and*

$$\nabla^{\tilde{t}}\left(\neg q\right)\left(\omega \right)=\{\begin{array}{cc} 0, & if\;\nabla (\neg q)(\omega )>0\\ 0, & if\;\nabla (\neg q)(\omega )=0\;and\;\Delta (q)(\omega )<N-1\\ N-1, & otherwise \end{array}$$

*for each q* ∈ *Q*_1_ *and ω* ∈ Ω.

**Definition 35**. *An N-BHS bottom weak complement of* (Δ, ∇, *Q*_1_, *N*) *is* ${\left(\Delta ,\nabla ,Q_{1},N\right)}^{\tilde{b}}=\left(\Delta^{\tilde{b}},\nabla^{\tilde{b}},Q_{1},N\right)$, *where*

$$\Delta^{\tilde{b}}\left(q\right)\left(\omega \right)=\{\begin{array}{cc} 0, & if\;\Delta (q)(\omega )>0\\ 0, & if\;\Delta (q)(\omega )=0\;and\;\nabla (\neg q)(\omega )<N-1\\ N-1, & otherwise \end{array}$$

*and*

$$\nabla^{\tilde{b}}\left(\neg q\right)\left(\omega \right)=\{\begin{array}{cc} N-1, & if\;\nabla (\neg q)(\omega )<N-1\\ 0, & if\;\nabla (\neg q)(\omega )=N-1 \end{array}$$

*for each q* ∈ *Q*_1_ *and ω* ∈ Ω.

**Remark 4**. *For every value of* 0 < *T* < *N, there is a corresponding BHS set associated with each N-BHS set*.

**Definition 36**. *For a given threshold* 0 < *T* < *N and an N-BHS set* (Δ, ∇, *Q*_1_, *N*), *the associated BHS set is denoted as* $\left(\Delta^{\tilde{T}},\nabla^{\tilde{T}},Q_{1}\right)$ *and is defined by the following expressions, for all q* ∈ *Q*_1_ *and ω* ∈ Ω,

$$\Delta^{\tilde{T}}\left(q\right)\left(\omega \right)=\{\begin{array}{cc} 1, & if\;\Delta (q)(\omega )\geq T\\ 0, & otherwise \end{array}$$

*and*

$$\nabla^{\tilde{T}}\left(\neg q\right)\left(\omega \right)=\{\begin{array}{cc} 1, & if\;\nabla (\neg q)(\omega )\geq N-T\\ 0, & otherwise \end{array}$$

Specifically, we define $\left(\Delta^{\tilde{T}=1},\nabla^{\tilde{T}=1},Q_{1}\right)$ as the bottom BHS set related to (Δ, ∇, *Q*_1_, *N*), while $\left(\Delta^{\tilde{T}=N-1},\nabla^{\tilde{T}=N-1},Q_{1}\right)$ is referred to as the top BHS set associated with (Δ, ∇, *Q*_1_, *N*).

## 4 Set-theoretic operations on *N*-bipolar hypersoft sets and their properties

In this section, the focus shifts towards investigating set-theoretic operations within the framework of *N*-BHS sets. These operations are introduced first, followed by a discussion on their pertinent properties and implications.

**Definition 37**. *An N-BHS set* $\left(\tilde{\Phi },\tilde{\Omega },Q_{1},N\right)$ *is referred to as a relative null N-BHS set if, for all q* ∈ *Q*_1_ *and ω* ∈ Ω, $\tilde{\Phi }\left(q\right)\left(\omega \right)=0$ *and* $\tilde{\Omega }\left(\neg q\right)\left(\omega \right)=N-1$.

**Definition 38**. *An N-BHS set* $\left(\tilde{\Omega },\tilde{\Phi },Q_{1},N\right)$ *is referred to as a relative whole N-BHS set if, for all q* ∈ *Q*_1_ *and ω* ∈ Ω, $\tilde{\Omega }\left(q\right)\left(\omega \right)=N-1$ *and* $\tilde{\Phi }\left(\neg q\right)\left(\omega \right)=0$.

**Definition 39**. *An N-BHS set* (Δ, ∇, *Q*_1_, *N*) *is defined as the N-BHS subset of* (Δ_1_, ∇_1_, *Q*_2_, *N*), *denoted by* (Δ, ∇, *Q*_1_, *N*) $\tilde{⊑}$ (Δ_1_, ∇_1_, *Q*_2_, *N*), *if the following conditions hold*:

1. *Q*_1_ ⊆ *Q*_2_.
2. Δ(*q*)(*ω*) ≤ Δ_1_(*q*)(*ω*) *and* ∇_1_(¬*q*)(*ω*) ≤ ∇(¬*q*)(*ω*) *for all q* ∈ *Q*_1_ *and ω* ∈ Ω.

**Definition 40**. *An N-BHS extended union of* (Δ, ∇, *Q*_1_, *N*_1_) *and* (Δ_1_, ∇_1_, *Q*_2_, *N*_2_) *is denoted and defined as* (Δ, ∇, *Q*_1_, *N*_1_) ${\tilde{⊔}}_{\varepsilon }$ (Δ_1_, ∇_1_, *Q*_2_, *N*_2_) = (Δ_2_, ∇_2_, *Q*_1_ ∪ *Q*_2_, *max*(*N*_1_, *N*_2_)), *where for all q* ∈ *Q*_1_ ∪ *Q*_2_ *and ω* ∈ Ω:

$$\Delta_{2}\left(q\right)\left(\omega \right)=\{\begin{array}{cc} \Delta (q)(\omega ) & if\;q\in Q_{1}\backslash Q_{2}\\ \Delta_{1}\left(q\right)\left(\omega \right) & if\;q\in Q_{2}\backslash Q_{1}\\ max\{\Delta \left(q\right)\left(\omega \right),\Delta_{1}\left(q\right)\left(\omega \right)\} & if\;q\in Q_{1}\cap Q_{2} \end{array}$$

*and*

$$\nabla_{2}\left(\neg q\right)\left(\omega \right)=\{\begin{array}{cc} \nabla (\neg q)(\omega ) & if\;\neg q\in \neg Q_{1}\backslash \neg Q_{2}\\ \nabla_{1}\left(\neg q\right)\left(\omega \right) & if\;\neg q\in \neg Q_{2}\backslash \neg Q_{1}\\ min\{\nabla \left(\neg q\right)\left(\omega \right),\nabla_{1}\left(\neg q\right)\left(\omega \right)\} & if\;\neg q\in \neg Q_{1}\cap \neg Q_{2} \end{array}$$

**Definition 41**. *An N-BHS extended intersection of* (Δ, ∇, *Q*_1_, *N*_1_) *and* (Δ_1_, ∇_1_, *Q*_2_, *N*_2_) *is denoted and defined as* (Δ, ∇, *Q*_1_, *N*_1_) ${\tilde{⊓}}_{\varepsilon }$ (Δ_1_, ∇_1_, *Q*_2_, *N*_2_) = (Δ_2_, ∇_2_, *Q*_1_ ∪ *Q*_2_, *max*(*N*_1_, *N*_2_)), *where for all q* ∈ *Q*_1_ ∪ *Q*_2_ *and ω* ∈ Ω:

$$\Delta_{2}\left(q\right)\left(\omega \right)=\{\begin{array}{cc} \Delta (q)(\omega ) & if\;q\in Q_{1}\backslash Q_{2}\\ \Delta_{1}\left(q\right)\left(\omega \right) & if\;q\in Q_{2}\backslash Q_{1}\\ min\{\Delta \left(q\right)\left(\omega \right),\Delta_{1}\left(q\right)\left(\omega \right)\} & if\;q\in Q_{1}\cap Q_{2} \end{array}$$

*and*

$$\nabla_{2}\left(\neg q\right)\left(\omega \right)=\{\begin{array}{cc} \nabla (\neg q)(\omega ) & if\;\neg q\in \neg Q_{1}\backslash \neg Q_{2}\\ \nabla_{1}\left(\neg q\right)\left(\omega \right) & if\;\neg q\in \neg Q_{2}\backslash \neg Q_{1}\\ max\{\nabla \left(\neg q\right)\left(\omega \right),\nabla_{1}\left(\neg q\right)\left(\omega \right)\} & if\;\neg q\in \neg Q_{1}\cap \neg Q_{2} \end{array}$$

**Definition 42**. *An N-BHS restricted union of* (Δ, ∇, *Q*_1_, *N*_1_) *and* (Δ_1_, ∇_1_, *Q*_2_, *N*_2_) *is denoted and defined as* (Δ, ∇, *Q*_1_, *N*_1_) ${\tilde{⊔}}_{ℜ}$ (Δ_1_, ∇_1_, *Q*_2_, *N*_2_) = (Δ_2_, ∇_2_, *Q*_1_ ∩ *Q*_2_, *max*(*N*_1_, *N*_2_)), *where for all q* ∈ *Q*_1_ ∩ *Q*_2_ ≠ ∅ *and ω* ∈ Ω: Δ_2_(*q*)(*ω*) = *max*{Δ(*q*)(*ω*), Δ_1_(*q*)(*ω*)} *and* ∇_2_(¬*q*)(*ω*) = *min* {∇(¬*q*)(*ω*), ∇_1_(¬*q*)(*ω*)}.

**Definition 43**. *An N-BHS restricted intersection of* (Δ, ∇, *Q*_1_, *N*_1_) *and* (Δ_1_, ∇_1_, *Q*_2_, *N*_2_) *is denoted and defined as* (Δ, ∇, *Q*_1_, *N*_1_) ${\tilde{⊓}}_{ℜ}$ (Δ_1_, ∇_1_, *Q*_2_, *N*_2_) = (Δ_2_, ∇_2_, *Q*_1_ ∩ *Q*_2_, *max*(*N*_1_, *N*_2_)), *where for all q* ∈ *Q*_1_ ∩ *Q*_2_ ≠ ∅ *and ω* ∈ Ω: Δ_2_(*q*)(*ω*) = *min*{Δ(*q*)(*ω*), Δ_1_(*q*)(*ω*)} *and* ∇_2_(¬*q*)(*ω*) = *max* {∇(¬*q*)(*ω*), ∇_1_(¬*q*)(*ω*)}.

**Example 4**. *Consider Example 3 and the two N-BHS sets* (Δ, ∇, *Q*_1_, 6) *and* (Δ_1_, ∇_1_, *Q*_2_, 7) *given in* Tables 6 *and* 7. *The N-BHS extended union (intersection) and the N-BHS restricted union (intersection) are given in* Tables 8–11.

**Table 6:** The 6-BHS set (Δ, ∇, *Q*_1_, 6) in Example 4.

(Δ, ∇, *Q*_1_, 6)	*q*_1_	*q*_2_	*q*_3_
*ω*_1_	(5,0)	(3,1)	(2,3)
*ω*_2_	(4,1)	(3,0)	(1,3)
*ω*_3_	(4,1)	(4,0)	(0,4)

**Table 7:** The 7-BHS set (Δ_1_, ∇_1_, *Q*_2_, 7) in Example 4.

(Δ_1_, ∇_1_, *Q*_2_, 7)	*q*_1_	*q*_2_	*q*_4_
*ω*_1_	(6,0)	(1,2)	(3,2)
*ω*_2_	(4,2)	(0,6)	(6,0)
*ω*_3_	(0,3)	(5,1)	(2,4)

**Table 8:** The *N*-BHS extended union of (Δ, ∇, *Q*_1_, 6) and (Δ_1_, ∇_1_, *Q*_2_, 7) in Example 4.

(Δ, ∇, *Q*_1_, 6) ${\tilde{⊔}}_{\varepsilon }$ (Δ_1_, ∇_1_, *Q*_2_, 7)	*q*_1_	*q*_2_	*q*_3_	*q*_4_
*ω*_1_	(6,0)	(3,1)	(2,3)	(3,2)
*ω*_2_	(4,1)	(3,0)	(1,3)	(6,0)
*ω*_3_	(4,1)	(5,0)	(0,4)	(2,4)

**Table 9:** The *N*-BHS extended intersection of (Δ, ∇, *Q*_1_, 6) and (Δ_1_, ∇_1_, *Q*_2_, 7) in Example 4.

(Δ, ∇, *Q*_1_, 6) ${\tilde{⊓}}_{\varepsilon }$ (Δ_1_, ∇_1_, *Q*_2_, 7)	*q*_1_	*q*_2_	*q*_3_	*q*_4_
*ω*_1_	(5,0)	(1,2)	(2,3)	(3,2)
*ω*_2_	(4,2)	(0,6)	(1,3)	(6,0)
*ω*_3_	(0,3)	(4,1)	(0,4)	(2,4)

**Table 10:** The *N*-BHS restricted union of (Δ, ∇, *Q*_1_, 6) and (Δ_1_, ∇_1_, *Q*_2_, 7) in Example 4.

(Δ, ∇, *Q*_1_, 6) ${\tilde{⊔}}_{ℜ}$ (Δ_1_, ∇_1_, *Q*_2_, 7)	*q*_1_	*q*_2_
*ω*_1_	(6,0)	(3,1)
*ω*_2_	(4,1)	(3,0)
*ω*_3_	(4,1)	(5,0)

**Table 11:** The *N*-BHS restricted intersection of (Δ, ∇, *Q*_1_, 6) and (Δ_1_, ∇_1_, *Q*_2_, 7) in Example 4.

(Δ, ∇, *Q*_1_, 6) ${\tilde{⊓}}_{ℜ}$ (Δ_1_, ∇_1_, *Q*_2_, 7)	*q*_1_	*q*_2_
*ω*_1_	(5,0)	(1,2)
*ω*_2_	(4,2)	(0,6)
*ω*_3_	(0,3)	(4,1)

**Definition 44**. *The N-BHS extended T-union of* (Δ, ∇, *Q*_1_, *N*_1_) *and* (Δ_1_, ∇_1_, *Q*_2_, *N*_2_) *where T* < *min*(*N*_1_, *N*_2_) *is defined as the BHS extended union of two BHS sets* $\left(\Delta^{\tilde{T}},\nabla^{\tilde{T}},Q_{1}\right)$ *and* $\left(\Delta_{1}^{\tilde{T}},\nabla_{1}^{\tilde{T}},Q_{2}\right)$. *It is denoted by* (Δ, ∇, *Q*_1_, *N*_1_) ${\tilde{⊔}}_{\varepsilon }^{\tilde{T}}$ (Δ_1_, ∇_1_, *Q*_2_, *N*_2_) = $\left(\Delta^{\tilde{T}},\nabla^{\tilde{T}},Q_{1}\right)$ ⊔_*e*_ $\left(\Delta_{1}^{\tilde{T}},\nabla_{1}^{\tilde{T}},Q_{2}\right)$.

**Definition 45**. *The N-BHS extended T-intersection of* (Δ, ∇, *Q*_1_, *N*_1_) *and* (Δ_1_, ∇_1_, *Q*_2_, *N*_2_) *where T* < *min*(*N*_1_, *N*_2_) *is defined as the BHS extended intersection of two BHS sets* $\left(\Delta^{\tilde{T}},\nabla^{\tilde{T}},Q_{1}\right)$ *and* $\left(\Delta_{1}^{\tilde{T}},\nabla_{1}^{\tilde{T}},Q_{2}\right)$. *It is denoted by* (Δ, ∇, *Q*_1_, *N*_1_) ${\tilde{⊓}}_{\varepsilon }^{\tilde{T}}(\Delta_{1},\nabla_{1},Q_{2},N_{2})=\left(\Delta^{\tilde{T}},\nabla^{\tilde{T}},Q_{1}\right){⊓}_{e}\left(\Delta_{1}^{\tilde{T}},\nabla_{1}^{\tilde{T}},Q_{2}\right)$.

**Definition 46**. *The N-BHS restricted T-union of* (Δ, ∇, *Q*_1_, *N*_1_) *and* (Δ_1_, ∇_1_, *Q*_2_, *N*_2_) *where T* < *min*(*N*_1_, *N*_2_) *is defined as the BHS restricted union of two BHS sets* $\left(\Delta^{\tilde{T}},\nabla^{\tilde{T}},Q_{1}\right)$ *and* $\left(\Delta_{1}^{\tilde{T}},\nabla_{1}^{\tilde{T}},Q_{2}\right)$. *It is denoted by* (Δ, ∇, *Q*_1_, *N*_1_) ${\tilde{⊔}}_{ℜ}^{\tilde{T}}(\Delta_{1},\nabla_{1},Q_{2},N_{2})=\left(\Delta^{\tilde{T}},\nabla^{\tilde{T}},Q_{1}\right)$ ⊔_*r*_ $\left(\Delta_{1}^{\tilde{T}},\nabla_{1}^{\tilde{T}},Q_{2}\right)$.

**Definition 47**. *The N-BHS restricted T-intersection of* (Δ, ∇, *Q*_1_, *N*_1_) *and* (Δ_1_, ∇_1_, *Q*_2_, *N*_2_) *where T* < *min*(*N*_1_, *N*_2_) *is defined as the BHS restricted intersection of two BHS sets* $\left(\Delta^{\tilde{T}},\nabla^{\tilde{T}},Q_{1}\right)$ *and* $\left(\Delta_{1}^{\tilde{T}},\nabla_{1}^{\tilde{T}},Q_{2}\right)$. *It is denoted by* (Δ, ∇, *Q*_1_, *N*_1_) ${\tilde{⊓}}_{ℜ}^{\tilde{T}}(\Delta_{1},\nabla_{1},Q_{2},N_{2})=\left(\Delta^{\tilde{T}},\nabla^{\tilde{T}},Q_{1}\right)$ ⊓_*r*_ $\left(\Delta_{1}^{\tilde{T}},\nabla_{1}^{\tilde{T}},Q_{2}\right)$.

**Proposition 1**. *Let* (Δ, ∇, *Q*_1_, *N*), (Δ_1_, ∇_1_, *Q*_1_, *N*), *and* (Δ_2_, ∇_2_, *Q*_1_, *N*) *be three N-BHS sets. Then*,

1. (Δ, ∇, *Q*_1_, *N*) $\tilde{⊑}\left(\tilde{\Omega },\tilde{\Phi },Q_{1},N\right)$.
2.
$\left(\tilde{\Phi },\tilde{\Omega },Q_{1},N\right)\tilde{⊑}$ (Δ, ∇, *Q*_1_, *N*).
3. *If* (Δ, ∇, *Q*_1_, *N*) $\tilde{⊑}$ (Δ_1_, ∇_1_, *Q*_1_, *N*) *and* (Δ_1_, ∇_1_, *Q*_1_, *N*) $\tilde{⊑}$ (Δ_2_, ∇_2_, *Q*_1_, *N*), *then* (Δ, ∇, *Q*_1_, *N*) $\tilde{⊑}$ (Δ_2_, ∇_2_, *Q*_1_, *N*).

*Proof*. Straightforward.

**Proposition 2**. *Let* (Δ, ∇, *Q*_1_, *N*) *and* (Δ_1_, ∇_1_, *Q*_2_, *N*) *be two N-BHS sets. Then*,

1. (Δ, ∇, *Q*_1_, *N*) ${\tilde{⊔}}_{\varepsilon }$ (Δ_1_, ∇_1_, *Q*_2_, *N*) *is the smallest N-BHS set which contains both* (Δ, ∇, *Q*_1_, *N*) *and* (Δ_1_, ∇_1_, *Q*_2_, *N*).
2. (Δ, ∇, *Q*_1_, *N*) ${\tilde{⊓}}_{ℜ}$ (Δ_1_, ∇_1_, *Q*_2_, *N*) *is the largest N-BHS set which is contained in both* (Δ, ∇, *Q*_1_, *N*) *and* (Δ_1_, ∇_1_, *Q*_2_, *N*).

*Proof*. Straightforward.

**Proposition 3**. *Let* (Δ, ∇, *Q*_1_, *N*) *and* (Δ_1_, ∇_1_, *Q*_1_, *N*) *be two N-BHS sets. Then*,

1.
${\left(\tilde{\Phi },\tilde{\Omega },Q_{1},N\right)}^{\tilde{c}}=\left(\tilde{\Omega },\tilde{\Phi },Q_{1},N\right)$ , ${\left(\tilde{\Phi },\tilde{\Omega },Q_{1},N\right)}^{\tilde{t}}=\left(\tilde{\Omega },\tilde{\Phi },Q_{1},N\right)$, *and* ${\left(\tilde{\Phi },\tilde{\Omega },Q_{1},N\right)}^{\tilde{b}}=\left(\tilde{\Omega },\tilde{\Phi },Q_{1},N\right)$.
2.
${\left(\tilde{\Omega },\tilde{\Phi },Q_{1},N\right)}^{\tilde{c}}=\left(\tilde{\Phi },\tilde{\Omega },Q_{1},N\right)$ , ${\left(\tilde{\Omega },\tilde{\Phi },Q_{1},N\right)}^{\tilde{t}}=\left(\tilde{\Phi },\tilde{\Omega },Q_{1},N\right)$, *and* ${\left(\tilde{\Omega },\tilde{\Phi },Q_{1},N\right)}^{\tilde{b}}=\left(\tilde{\Phi },\tilde{\Omega },Q_{1},N\right)$.
3.
${\left({\left(\Delta ,\nabla ,Q_{1},N\right)}^{\tilde{c}}\right)}^{\tilde{c}}$ = (Δ, ∇, *Q*_1_, *N*), ${\left({\left(\Delta ,\nabla ,Q_{1},N\right)}^{\tilde{t}}\right)}^{\tilde{t}}\tilde{⊑}$ (Δ, ∇, *Q*_1_, *N*), *and* (Δ, ∇, *Q*_1_, *N*) $\tilde{⊑}{\left({\left(\Delta ,\nabla ,Q_{1},N\right)}^{\tilde{b}}\right)}^{\tilde{b}}$.
4. If (Δ, ∇, *Q*_1_, *N*) $\tilde{⊑}$ (Δ_1_, ∇_1_, *Q*_1_, *N*), *then* ${\left(\Delta_{1},\nabla_{1},Q_{1},N\right)}^{\tilde{c}}\tilde{⊑}{\left(\Delta ,\nabla ,Q_{1},N\right)}^{\tilde{c}}$, ${\left(\Delta_{1},\nabla_{1},Q_{1},N\right)}^{\tilde{t}}\tilde{⊑}{\left(\Delta ,\nabla ,Q_{1},N\right)}^{\tilde{t}}$, *and* ${\left(\Delta_{1},\nabla_{1},Q_{1},N\right)}^{\tilde{b}}\tilde{⊑}{\left(\Delta ,\nabla ,Q_{1},N\right)}^{\tilde{b}}$.
5.
$\left(\tilde{\Phi },\tilde{\Omega },Q_{1},N\right)\tilde{⊑}$ (Δ, ∇, *Q*_1_, *N*) ${\tilde{⊓}}_{ℜ}{\left(\Delta ,\nabla ,Q_{1},N\right)}^{\tilde{c}}\tilde{⊑}$ (Δ, ∇, *Q*_1_, *N*) ${\tilde{⊔}}_{ℜ}{\left(\Delta ,\nabla ,Q_{1},N\right)}^{\tilde{c}}\tilde{⊑}\left(\tilde{\Omega },\tilde{\Phi },Q_{1},N\right)$.
6. (Δ, ∇, *Q*_1_, *N*) ${\tilde{⊓}}_{ℜ}{\left(\Delta ,\nabla ,Q_{1},N\right)}^{\tilde{b}}=\left(\tilde{\Phi },\tilde{\Omega },Q_{1},N\right)$.
7. (Δ, ∇, *Q*_1_, *N*) ${\tilde{⊔}}_{ℜ}{\left(\Delta ,\nabla ,Q_{1},N\right)}^{\tilde{t}}=\left(\tilde{\Omega },\tilde{\Phi },Q_{1},N\right)$.
8. *If* (Δ, ∇, *Q*_1_, *N*) $\tilde{⊑}$ (Δ_1_, ∇_1_, *Q*_1_, *N*), *then* (Δ, ∇, *Q*_1_, *N*) ${\tilde{⊓}}_{ℜ}$ (Δ_1_, ∇_1_, *Q*_1_, *N*) = (Δ, ∇, *Q*_1_, *N*).
9. *If* (Δ, ∇, *Q*_1_, *N*) $\tilde{⊑}$ (Δ_1_, ∇_1_, *Q*_1_, *N*), *then* (Δ, ∇, *Q*_1_, *N*) ${\tilde{⊔}}_{ℜ}$ (Δ_1_, ∇_1_, *Q*_1_, *N*) = (Δ_1_, ∇_1_, *Q*_1_, *N*).

*Proof*. Straightforward.

**Proposition 4**. *Let* (Δ, ∇, *Q*_1_, *N*) *and* (Δ_1_, ∇_1_, *Q*_2_, *N*) *be two N-BHS sets. Then*,

1. ((Δ, ∇, *Q*_1_, *N*) ${\tilde{⊔}}_{\varepsilon }\left(\Delta_{1},\nabla_{1},Q_{2},N\right){)}^{\tilde{c}}={\left(\Delta ,\nabla ,Q_{1},N\right)}^{\tilde{c}}{\tilde{⊓}}_{\varepsilon }{\left(\Delta_{1},\nabla_{1},Q_{2},N\right)}^{\tilde{c}}$.
2. ((Δ, ∇, *Q*_1_, *N*) ${\tilde{⊓}}_{\varepsilon }\left(\Delta_{1},\nabla_{1},Q_{2},N\right){)}^{\tilde{c}}={\left(\Delta ,\nabla ,Q_{1},N\right)}^{\tilde{c}}{\tilde{⊔}}_{\varepsilon }{\left(\Delta_{1},\nabla_{1},Q_{2},N\right)}^{\tilde{c}}$.
3. ((Δ, ∇, *Q*_1_, *N*) ${\tilde{⊔}}_{\varepsilon }\left(\Delta_{1},\nabla_{1},Q_{2},N\right){)}^{\tilde{t}}={\left(\Delta ,\nabla ,Q_{1},N\right)}^{\tilde{t}}{\tilde{⊓}}_{\varepsilon }{\left(\Delta_{1},\nabla_{1},Q_{2},N\right)}^{\tilde{t}}$.
4. ((Δ, ∇, *Q*_1_, *N*) ${\tilde{⊓}}_{\varepsilon }\left(\Delta_{1},\nabla_{1},Q_{2},N\right){)}^{\tilde{t}}={\left(\Delta ,\nabla ,Q_{1},N\right)}^{\tilde{t}}{\tilde{⊔}}_{\varepsilon }{\left(\Delta_{1},\nabla_{1},Q_{2},N\right)}^{\tilde{t}}$.
5. ((Δ, ∇, *Q*_1_, *N*) ${\tilde{⊔}}_{\varepsilon }\left(\Delta_{1},\nabla_{1},Q_{2},N\right){)}^{\tilde{b}}={\left(\Delta ,\nabla ,Q_{1},N\right)}^{\tilde{b}}{\tilde{⊓}}_{\varepsilon }{\left(\Delta_{1},\nabla_{1},Q_{2},N\right)}^{\tilde{b}}$.
6. ((Δ, ∇, *Q*_1_, *N*) ${\tilde{⊓}}_{\varepsilon }\left(\Delta_{1},\nabla_{1},Q_{2},N\right){)}^{\tilde{b}}={\left(\Delta ,\nabla ,Q_{1},N\right)}^{\tilde{b}}{\tilde{⊔}}_{\varepsilon }{\left(\Delta_{1},\nabla_{1},Q_{2},N\right)}^{\tilde{b}}$.
7. ((Δ, ∇, *Q*_1_, *N*) ${\tilde{⊔}}_{ℜ}\left(\Delta_{1},\nabla_{1},Q_{2},N\right){)}^{\tilde{c}}={\left(\Delta ,\nabla ,Q_{1},N\right)}^{\tilde{c}}{\tilde{⊓}}_{ℜ}{\left(\Delta_{1},\nabla_{1},Q_{2},N\right)}^{\tilde{c}}$.
8. ((Δ, ∇, *Q*_1_, *N*) ${\tilde{⊓}}_{ℜ}\left(\Delta_{1},\nabla_{1},Q_{2},N\right){)}^{\tilde{c}}={\left(\Delta ,\nabla ,Q_{1},N\right)}^{\tilde{c}}{\tilde{⊔}}_{ℜ}{\left(\Delta_{1},\nabla_{1},Q_{2},N\right)}^{\tilde{c}}$.
9. ((Δ, ∇, *Q*_1_, *N*) ${\tilde{⊔}}_{ℜ}\left(\Delta_{1},\nabla_{1},Q_{2},N\right){)}^{\tilde{t}}={\left(\Delta ,\nabla ,Q_{1},N\right)}^{\tilde{t}}{\tilde{⊓}}_{ℜ}{\left(\Delta_{1},\nabla_{1},Q_{2},N\right)}^{\tilde{t}}$.
10. ((Δ, ∇, *Q*_1_, *N*) ${\tilde{⊓}}_{ℜ}\left(\Delta_{1},\nabla_{1},Q_{2},N\right){)}^{\tilde{t}}={\left(\Delta ,\nabla ,Q_{1},N\right)}^{\tilde{t}}{\tilde{⊔}}_{ℜ}{\left(\Delta_{1},\nabla_{1},Q_{2},N\right)}^{\tilde{t}}$.
11. ((Δ, ∇, *Q*_1_, *N*) ${\tilde{⊔}}_{ℜ}\left(\Delta_{1},\nabla_{1},Q_{2},N\right){)}^{\tilde{b}}={\left(\Delta ,\nabla ,Q_{1},N\right)}^{\tilde{b}}{\tilde{⊓}}_{ℜ}{\left(\Delta_{1},\nabla_{1},Q_{2},N\right)}^{\tilde{b}}$.
12. ((Δ, ∇, *Q*_1_, *N*) ${\tilde{⊓}}_{ℜ}\left(\Delta_{1},\nabla_{1},Q_{2},N\right){)}^{\tilde{b}}={\left(\Delta ,\nabla ,Q_{1},N\right)}^{\tilde{b}}{\tilde{⊔}}_{ℜ}{\left(\Delta_{1},\nabla_{1},Q_{2},N\right)}^{\tilde{b}}$.

*Proof*. (3) Suppose that (Δ, ∇, *Q*_1_, *N*) ${\tilde{⊔}}_{\varepsilon }$ (Δ_1_, ∇_1_, *Q*_2_, *N*) = (Δ_2_, ∇_2_, *Q*_1_ ∪ *Q*_2_, *N*). Then, for all *q* ∈ *Q*_1_ ∪ *Q*_2_ and *ω* ∈ Ω:

$$\Delta_{2}\left(q\right)\left(\omega \right)=\{\begin{array}{cc} \Delta (q)(\omega ) & \text{if}\;q\in Q_{1}\backslash Q_{2}\\ \Delta_{1}\left(q\right)\left(\omega \right) & \text{if}\;q\in Q_{2}\backslash Q_{1}\\ max\{\Delta \left(q\right)\left(\omega \right),\Delta_{1}\left(q\right)\left(\omega \right)\} & \text{if}\;q\in Q_{1}\cap Q_{2} \end{array}$$

and

$$\nabla_{2}\left(\neg q\right)\left(\omega \right)=\{\begin{array}{cc} \nabla (\neg q)(\omega ) & \text{if}\;\neg q\in \neg Q_{1}\backslash \neg Q_{2}\\ \nabla_{1}\left(\neg q\right)\left(\omega \right) & \text{if}\;\neg q\in \neg Q_{2}\backslash \neg Q_{1}\\ min\{\nabla \left(\neg q\right)\left(\omega \right),\nabla_{1}\left(\neg q\right)\left(\omega \right)\} & \text{if}\;\neg q\in \neg Q_{1}\cap \neg Q_{2} \end{array}$$

Now, ((Δ, ∇, *Q*_1_, *N*) ${\tilde{⊔}}_{\varepsilon }\left(\Delta_{1},\nabla_{1},Q_{2},N\right){)}^{\tilde{t}}={\left(\Delta_{2},\nabla_{2},Q_{1}\cup Q_{2},N\right)}^{\tilde{t}}$. Then, for all *q* ∈ *Q*_1_ ∪ *Q*_2_ and *ω* ∈ Ω:

$$\Delta_{2}^{\tilde{t}}\left(q\right)\left(\omega \right)=\{\begin{array}{cc} N-1, & \text{if}\;\Delta (q)(\omega )<N-1\;\text{and}\;q\in Q_{1}\backslash Q_{2}\\ 0, & \text{if}\;\Delta (q)(\omega )=N-1\;\text{and}\;q\in Q_{1}\backslash Q_{2}\\ N-1, & \text{if}\;\Delta_{1}\left(q\right)\left(\omega \right)<N-1\;\text{and}\;q\in Q_{2}\backslash Q_{1}\\ 0, & \text{if}\;\Delta_{1}\left(q\right)\left(\omega \right)=N-1\;\text{and}\;q\in Q_{2}\backslash Q_{1}\\ N-1, & \text{if}\;max\;\{\Delta \left(q\right)\left(\omega \right),\Delta_{1}\left(q\right)\left(\omega \right)\}<N-1\;\text{and}\;q\in Q_{1}\cap Q_{2}\\ 0, & \text{if}\;max\;\{\Delta \left(q\right)\left(\omega \right),\Delta_{1}\left(q\right)\left(\omega \right)\}=N-1\;\text{and}\;q\in Q_{1}\cap Q_{2} \end{array}$$

$$=\{\begin{array}{cc} N-1, & \text{if}\;\Delta \left(q\right)\left(\omega \right)<N-1\;\text{and}\;q\in Q_{1}\backslash Q_{2}\\ 0, & \text{if}\;\Delta \left(q\right)\left(\omega \right)=N-1\;\text{and}\;q\in Q_{1}\backslash Q_{2}\\ N-1, & \text{if}\;\Delta_{1}\left(q\right)\left(\omega \right)<N-1\;\text{and}\;q\in Q_{2}\backslash Q_{1}\\ 0, & \text{if}\;\Delta_{1}\left(q\right)\left(\omega \right)=N-1\;\text{and}\;q\in Q_{2}\backslash Q_{1}\\ N-1, & \text{if}\;\Delta \left(q\right)\left(\omega \right)<N-1\;\text{and}\;\Delta_{1}\left(q\right)\left(\omega \right)<N-1\;\text{and}\;q\in Q_{1}\cap Q_{2}\\ 0, & \text{if}\;\Delta \left(q\right)\left(\omega \right)=N-1\;\text{and}\;\Delta_{1}\left(q\right)\left(\omega \right)=N-1\\ & \;\text{or}\;\Delta \left(q\right)\left(\omega \right)<N-1\;\text{and}\;\Delta_{1}\left(q\right)\left(\omega \right)=N-1\\ & \;\text{or}\;\Delta \left(q\right)\left(\omega \right)=N-1\;\text{and}\;\Delta_{1}\left(q\right)\left(\omega \right)<N-1\\ & \;\text{and}\;q\in Q_{1}\cap Q_{2} \end{array}$$

and

$$\nabla_{2}^{\tilde{t}}\left(\neg q\right)\left(\omega \right)=\{\begin{array}{cc} 0, & \text{if}\;\nabla \left(\neg q\right)\left(\omega \right)>0\;\text{and}\;\neg q\in \neg Q_{1}\backslash \neg Q_{2}\\ 0, & \text{if}\;\nabla \left(\neg q\right)\left(\omega \right)=0\;\text{and}\;\Delta \left(q\right)\left(\omega \right)<N-1\;\text{and}\;\neg q\in \neg Q_{1}\backslash \neg Q_{2}\\ N-1, & \text{if}\;\nabla \left(\neg q\right)\left(\omega \right)=0\;\text{and}\;\neg q\in \neg Q_{1}\backslash \neg Q_{2}\\ 0, & \text{if}\;\nabla_{1}\left(\neg q\right)\left(\omega \right)>0\;\text{and}\;\neg q\in \neg Q_{2}\backslash \neg Q_{1}\\ 0, & \text{if}\;\nabla_{1}\left(\neg q\right)\left(\omega \right)=0\;\text{and}\;\Delta_{1}\left(q\right)\left(\omega \right)<N-1\;\text{and}\;\neg q\in \neg Q_{2}\backslash \neg Q_{1}\\ N-1, & \text{if}\;\nabla_{1}\left(\neg q\right)\left(\omega \right)=0\;\text{and}\;\neg q\in \neg Q_{2}\backslash \neg Q_{1}\\ 0, & \text{if}\;min\left\{\nabla \left(\neg q\right)\left(\omega \right),\nabla_{1}\left(\neg q\right)\left(\omega \right)\right\}>0\;\text{and}\;\neg q\in \neg Q_{1}\cap \neg Q_{2}\\ 0, & \text{if}\;min\left\{\nabla \left(\neg q\right)\left(\omega \right),\nabla_{1}\left(\neg q\right)\left(\omega \right)\right\}=0\;\text{and}\;max\left\{\Delta \left(q\right)\left(\omega \right),\Delta_{1}\left(q\right)\left(\omega \right)\right\}<N-1\\ & \;\text{and}\;\neg q\in \neg Q_{1}\cap \neg Q_{2}\\ N-1, & \text{if}\;min\left\{\nabla \left(\neg q\right)\left(\omega \right),\nabla_{1}\left(\neg q\right)\left(\omega \right)\right\}=0\;\text{and}\;\neg q\in \neg Q_{1}\cap \neg Q_{2} \end{array}$$

$$=\{\begin{array}{cc} 0, & \text{if}\;\nabla \left(\neg q\right)\left(\omega \right)>0\;\text{and}\;\neg q\in \neg Q_{1}\backslash \neg Q_{2}\\ 0, & \text{if}\;\nabla \left(\neg q\right)\left(\omega \right)=0\;\text{and}\;\Delta \left(q\right)\left(\omega \right)<N-1\;\text{and}\;\neg q\in \neg Q_{1}\backslash \neg Q_{2}\\ N-1, & \text{if}\;\nabla \left(\neg q\right)\left(\omega \right)=0\;\text{and}\;\neg q\in \neg Q_{1}\backslash \neg Q_{2}\\ 0, & \text{if}\;\nabla_{1}\left(\neg q\right)\left(\omega \right)>0\;\text{and}\;\neg q\in \neg Q_{2}\backslash \neg Q_{1}\\ 0, & \text{if}\;\nabla_{1}\left(\neg q\right)\left(\omega \right)=0\;\text{and}\;\Delta_{1}\left(q\right)\left(\omega \right)<N-1\;\text{and}\;\neg q\in \neg Q_{2}\backslash \neg Q_{1}\\ N-1, & \text{if}\;\nabla_{1}\left(\neg q\right)\left(\omega \right)=0\;\text{and}\;\neg q\in \neg Q_{2}\backslash \neg Q_{1}\\ 0, & \text{if}\;\nabla \left(\neg q\right)\left(\omega \right)>0\;\text{and}\;\nabla_{1}\left(\neg q\right)\left(\omega \right)>0\;\text{and}\;\neg q\in \neg Q_{1}\cap \neg Q_{2}\\ 0, & \text{if}\;\nabla \left(\neg q\right)\left(\omega \right)=0\;\text{and}\;\nabla_{1}\left(\neg q\right)\left(\omega \right)>0\;\text{and}\;\Delta \left(q\right)\left(\omega \right)<N-1,\\ & \;\text{or}\;\nabla \left(\neg q\right)\left(\omega \right)>0\;\text{and}\;\nabla_{1}\left(\neg q\right)\left(\omega \right)=0\;\text{and}\;\Delta_{1}\left(q\right)\left(\omega \right)<N-1,\\ & \;\text{or}\;\nabla \left(\neg q\right)\left(\omega \right)=0\;\text{when}\;\Delta \left(q\right)\left(\omega \right)<N-1\;\text{and}\;\nabla_{1}\left(\neg q\right)\left(\omega \right)=0\;\text{when}\;\Delta_{1}\left(q\right)\left(\omega \right)<N-1\\ & \;\text{and}\;\neg q\in \neg Q_{1}\cap \neg Q_{2}\\ N-1, & \text{if}\;\nabla \left(\neg q\right)\left(\omega \right)=0\;\text{and}\;\nabla_{1}\left(\neg q\right)\left(\omega \right)=0\\ & \;\text{or}\;\nabla \left(\neg q\right)\left(\omega \right)=0\;\text{or}\;\nabla_{1}\left(\neg q\right)\left(\omega \right)=0\\ & \;\text{or}\;\nabla_{1}\left(\neg q\right)\left(\omega \right)=0\;\text{and}\;\nabla \left(\neg q\right)\left(\omega \right)=0\;\text{and}\;\Delta \left(q\right)\left(\omega \right)<N-1\\ & \;\text{or}\;\nabla \left(\neg q\right)\left(\omega \right)=0\;\text{and}\;\nabla_{1}\left(\neg q\right)\left(\omega \right)=0\;\text{and}\;\Delta_{1}\left(q\right)\left(\omega \right)<N-1 \end{array}$$

On the other hand, let ${\left(\Delta ,\nabla ,Q_{1},N\right)}^{\tilde{t}}{\tilde{⊓}}_{\varepsilon }{\left(\Delta_{1},\nabla_{1},Q_{2},N\right)}^{\tilde{t}}$ = (Δ_3_, ∇_3_, *Q*_1_ ∪ *Q*_2_, *N*), then for all *q* ∈ *Q*_1_ ∪ *Q*_2_ and *ω* ∈ Ω:

$$\Delta_{3}\left(q\right)\left(\omega \right)=\{\begin{array}{cc} \Delta^{\tilde{t}}\left(q\right)\left(\omega \right) & \text{if}\;q\in Q_{1}\backslash Q_{2}\\ \Delta_{1}^{\tilde{t}}\left(q\right)\left(\omega \right) & \text{if}\;q\in Q_{2}\backslash Q_{1}\\ min\{\Delta^{\tilde{t}}\left(q\right)\left(\omega \right),\Delta_{1}^{\tilde{t}}\left(q\right)\left(\omega \right)\} & \text{if}\;q\in Q_{1}\cap Q_{2} \end{array}$$

and

$$\nabla_{3}\left(\neg q\right)\left(\omega \right)=\{\begin{array}{cc} \nabla^{\tilde{t}}\left(\neg q\right)\left(\omega \right) & \text{if}\;\neg q\in \neg Q_{1}\backslash \neg Q_{2}\\ \nabla_{1}^{\tilde{t}}\left(\neg q\right)\left(\omega \right) & \text{if}\;\neg q\in \neg Q_{2}\backslash \neg Q_{1}\\ max\{\nabla^{\tilde{t}}\left(\neg q\right)\left(\omega \right),\nabla_{1}^{\tilde{t}}\left(\neg q\right)\left(\omega \right)\} & \text{if}\;\neg q\in \neg Q_{1}\cap \neg Q_{2} \end{array}$$

Hence,

$$\Delta_{3}\left(q\right)\left(\omega \right)=\{\begin{array}{cc} N-1, & \text{if}\;\Delta (q)(\omega )<N-1\;\text{and}\;q\in Q_{1}\backslash Q_{2}\\ 0, & \text{if}\;\Delta (q)(\omega )=N-1\;\text{and}\;q\in Q_{1}\backslash Q_{2}\\ N-1, & \text{if}\;\Delta_{1}\left(q\right)\left(\omega \right)<N-1\;\text{and}\;q\in Q_{2}\backslash Q_{1}\\ 0, & \text{if}\;\Delta_{1}\left(q\right)\left(\omega \right)=N-1\;\text{and}\;q\in Q_{2}\backslash Q_{1}\\ N-1, & \text{if}\;min\;\{\Delta^{\tilde{t}}\left(q\right)\left(\omega \right),{\tilde{F}}_{1}^{\tilde{t}}\left(q\right)\left(\omega \right)\}<N-1\;\text{and}\;q\in Q_{1}\cap Q_{2}\\ 0, & \text{if}\;min\;\{\Delta^{\tilde{t}}\left(q\right)\left(\omega \right),{\tilde{F}}_{1}^{\tilde{t}}\left(q\right)\left(\omega \right)\}=N-1\;\text{and}\;q\in Q_{1}\cap Q_{2} \end{array}$$

$$\begin{array}{cc} = & \left\{ \begin{array}{cc} N-1, & \text{if}\;\Delta \left(q\right)\left(\omega \right)<N-1\;\text{and}\;q\in Q_{1}\backslash Q_{2}\\ 0, & \text{if}\;\Delta \left(q\right)\left(\omega \right)=N-1\;\text{and}\;q\in Q_{1}\backslash Q_{2}\\ N-1, & \text{if}\;\Delta_{1}\left(q\right)\left(\omega \right)<N-1\;\text{and}\;q\in Q_{2}\backslash Q_{1}\\ 0, & \text{if}\;\Delta_{1}\left(q\right)\left(\omega \right)=N-1\;\text{and}\;q\in Q_{2}\backslash Q_{1}\\ N-1, & \text{if}\;\Delta \left(q\right)\left(\omega \right)<N-1\;\text{and}\;\Delta_{1}\left(q\right)\left(\omega \right)<N-1\;\text{and}\;q\in Q_{1}\cap Q_{2}\\ 0, & \text{if}\;\Delta \left(q\right)\left(\omega \right)=N-1\;\text{and}\;\Delta_{1}\left(q\right)\left(\omega \right)=N-1\\ & \;\text{or}\;\Delta \left(q\right)\left(\omega \right)<N-1\;\text{and}\;\Delta_{1}\left(q\right)\left(\omega \right)=N-1\\ & \;\text{or}\;\Delta \left(q\right)\left(\omega \right)=N-1\;\text{and}\;\Delta_{1}\left(q\right)\left(\omega \right)<N-1\\ & \;\text{and}\;q\in Q_{1}\cap Q_{2} \end{array}\right. \end{array}$$

and

$$\nabla_{3}\left(\neg q\right)\left(\omega \right)=\{\begin{array}{cc} 0, & \text{if}\;\nabla \left(\neg q\right)\left(\omega \right)>0\;\text{and}\;\neg q\in \neg Q_{1}\backslash \neg Q_{2}\\ 0, & \text{if}\;\nabla \left(\neg q\right)\left(\omega \right)=0\;\text{and}\;\Delta \left(q\right)\left(\omega \right)<N-1\;\text{and}\;\neg q\in \neg Q_{1}\backslash \neg Q_{2}\\ N-1, & \text{if}\;\nabla \left(\neg q\right)\left(\omega \right)=0\;\text{and}\;\neg q\in \neg Q_{1}\backslash \neg Q_{2}\\ 0, & \text{if}\;\nabla_{1}\left(\neg q\right)\left(\omega \right)>0\;\text{and}\;\neg q\in \neg Q_{2}\backslash \neg Q_{1}\\ 0, & \text{if}\;\nabla_{1}\left(\neg q\right)\left(\omega \right)=0\;\text{and}\;\Delta_{1}\left(q\right)\left(\omega \right)<N-1\;\text{and}\;\neg q\in \neg Q_{2}\backslash \neg Q_{1}\\ N-1, & \text{if}\;\nabla_{1}\left(\neg q\right)\left(\omega \right)=0\;\text{and}\;\neg q\in \neg Q_{2}\backslash \neg Q_{1}\\ 0, & \text{if}\;max\left\{\nabla^{\tilde{t}}\left(\neg q\right)\left(\omega \right),\nabla_{1}^{\tilde{t}}\left(\neg q\right)\left(\omega \right)\right\}>0\;\text{and}\;\neg q\in \neg Q_{1}\cap \neg Q_{2}\\ 0, & \text{if}\;max\left\{\nabla^{\tilde{t}}\left(\neg q\right)\left(\omega \right),\nabla_{1}^{\tilde{t}}\left(\neg q\right)\left(\omega \right)\right\}=0\;\text{and}\;max\left\{\Delta \left(q\right)\left(\omega \right),\Delta_{1}\left(q\right)\left(\omega \right)\right\}<N-1\\ & \;\text{and}\;\neg q\in \neg Q_{1}\cap \neg Q_{2}\\ N-1, & \text{if}\;max\left\{\nabla^{\tilde{t}}\left(\neg q\right)\left(\omega \right),\nabla_{1}^{\tilde{t}}\left(\neg q\right)\left(\omega \right)\right\}=0\;\text{and}\;\neg q\in \neg Q_{1}\cap \neg Q_{2} \end{array}$$

$$=\left\{ \begin{array}{cc} 0, & \text{if}\;\nabla \left(\neg q\right)\left(\omega \right)>0\;\text{and}\;\neg q\in \neg Q_{1}\backslash \neg Q_{2}\\ 0, & \text{if}\;\nabla \left(\neg q\right)\left(\omega \right)=0\;\text{and}\;\Delta \left(q\right)\left(\omega \right)<N-1\;\text{and}\;\neg q\in \neg Q_{1}\backslash \neg Q_{2}\\ N-1, & \text{if}\;\nabla \left(\neg q\right)\left(\omega \right)=0\;\text{and}\;\neg q\in \neg Q_{1}\backslash \neg Q_{2}\\ 0, & \text{if}\;\nabla_{1}\left(\neg q\right)\left(\omega \right)>0\;\text{and}\;\neg q\in \neg Q_{2}\backslash \neg Q_{1}\\ 0, & \text{if}\;\nabla_{1}\left(\neg q\right)\left(\omega \right)=0\;\text{and}\;\Delta_{1}\left(q\right)\left(\omega \right)<N-1\;\text{and}\;\neg q\in \neg Q_{2}\backslash \neg Q_{1}\\ N-1, & \text{if}\;\nabla_{1}\left(\neg q\right)\left(\omega \right)=0\;\text{and}\;\neg q\in \neg Q_{2}\backslash \neg Q_{1}\\ 0, & \text{if}\;\nabla \left(\neg q\right)\left(\omega \right)>0\;\text{and}\;\nabla_{1}\left(\neg q\right)\left(\omega \right)>0\;\text{and}\;\neg q\in \neg Q_{1}\cap \neg Q_{2}\\ 0, & \text{if}\;\nabla \left(\neg q\right)\left(\omega \right)=0\;\text{and}\;\nabla_{1}\left(\neg q\right)\left(\omega \right)>0\;\text{and}\;\Delta \left(q\right)\left(\omega \right)<N-1,\\ & \;\text{or}\;\nabla \left(\neg q\right)\left(\omega \right)>0\;\text{and}\;\nabla_{1}\left(\neg q\right)\left(\omega \right)=0\;\text{and}\;\Delta_{1}\left(q\right)\left(\omega \right)<N-1,\\ & \;\text{or}\;\nabla \left(\neg q\right)\left(\omega \right)=0\;\text{when}\;\Delta \left(q\right)\left(\omega \right)<N-1\;\text{and}\;\nabla_{1}\left(\neg q\right)\left(\omega \right)=0\;\text{when}\;\Delta_{1}\left(q\right)\left(\omega \right)<N-1\\ & \;\text{and}\;\neg q\in \neg Q_{1}\cap \neg Q_{2}\\ N-1, & \text{if}\;\nabla \left(\neg q\right)\left(\omega \right)=0\;\text{and}\;\nabla_{1}\left(\neg q\right)\left(\omega \right)=0\\ & \;\text{or}\;\nabla \left(\neg q\right)\left(\omega \right)=0\;\text{or}\;\nabla_{1}\left(\neg q\right)\left(\omega \right)=0\\ & \;\text{or}\;\nabla_{1}\left(\neg q\right)\left(\omega \right)=0\;\text{and}\;\nabla \left(\neg q\right)\left(\omega \right)=0\;\text{and}\;\Delta \left(q\right)\left(\omega \right)<N-1\\ & \;\text{or}\;\nabla \left(\neg q\right)\left(\omega \right)=0\;\text{and}\;\nabla_{1}\left(\neg q\right)\left(\omega \right)=0\;\text{and}\;\Delta_{1}\left(q\right)\left(\omega \right)<N-1 \end{array}\right.$$

Since ${\left(\Delta_{2},\nabla_{2},Q_{1}\cup Q_{2},N\right)}^{\tilde{t}}$ and (Δ_3_, ∇_3_, *Q*_1_ ∪ *Q*_2_, *N*) are equivalent for all *q* ∈ *Q*_1_ ∪ *Q*_2_ and *ω* ∈ Ω, the proof is concluded.

The other parts can be proven in a similar manner.

**Proposition 5**. *Let* (Δ, ∇, *Q*_1_, *N*) *and* (Δ_1_, ∇_1_, *Q*_1_, *N*) *be two N-BHS sets. Then*,

1. (Δ, ∇, *Q*_1_, *N*) ${\tilde{⊔}}_{\varepsilon }$ (Δ_1_, ∇_1_, *Q*_1_, *N*) = (Δ, ∇, *Q*_1_, *N*) ${\tilde{⊔}}_{ℜ}$ (Δ_1_, ∇_1_, *Q*_1_, *N*).
2. (Δ, ∇, *Q*_1_, *N*) ${\tilde{⊓}}_{\varepsilon }$ (Δ_1_, ∇_1_, *Q*_1_, *N*) = (Δ, ∇, *Q*_1_, *N*) ${\tilde{⊓}}_{ℜ}$ (Δ_1_, ∇_1_, *Q*_1_, *N*).
3. (Δ, ∇, *Q*_1_, *N*) ${\tilde{⊔}}_{ℜ}$ (Δ, ∇, *Q*_1_, *N*) = (Δ, ∇, *Q*_1_, *N*) *and* (Δ, ∇, *Q*_1_, *N*) ${\tilde{⊓}}_{ℜ}$ (Δ, ∇, *Q*_1_, *N*) = (Δ, ∇, *Q*_1_, *N*).
4. (Δ, ∇, *Q*_1_, *N*) ${\tilde{⊔}}_{ℜ}\left(\tilde{\Phi },\tilde{\Omega },Q_{1},N\right)$ = (Δ, ∇, *Q*_1_, *N*) *and* (Δ, ∇, *Q*_1_, *N*) ${\tilde{⊓}}_{ℜ}\left(\tilde{\Phi },\tilde{\Omega },Q_{1},N\right)=\left(\tilde{\Phi },\tilde{\Omega },Q_{1},N\right)$.
5. (Δ, ∇, *Q*_1_, *N*) ${\tilde{⊔}}_{ℜ}\left(\tilde{\Omega },\tilde{\Phi },Q_{1},N\right)=\left(\tilde{\Omega },\tilde{\Phi },Q_{1},N\right)$ *and* (Δ, ∇, *Q*_1_, *N*) ${\tilde{⊓}}_{ℜ}\left(\tilde{\Omega },\tilde{\Phi },Q_{1},N\right)=(\Delta ,\nabla ,Q_{1},N)$.

*Proof*. Straightforward.

**Proposition 6**. *Let* (Δ, ∇, *Q*_1_, *N*_1_), (Δ_1_, ∇_1_, *Q*_2_, *N*_2_), *and* (Δ_2_, ∇_2_, *Q*_3_, *N*_3_) *be three N-BHS sets. Then*,

1. (Δ, ∇, *Q*_1_, *N*_1_) ${\tilde{⊔}}_{\varepsilon }$ (Δ_1_, ∇_1_, *Q*_2_, *N*_2_) = (Δ_1_, ∇_1_, *Q*_2_, *N*_2_) ${\tilde{⊔}}_{\varepsilon }$ (Δ, ∇, *Q*_1_, *N*_1_).
2. (Δ, ∇, *Q*_1_, *N*_1_) ${\tilde{⊓}}_{\varepsilon }$ (Δ_1_, ∇_1_, *Q*_2_, *N*_2_) = (Δ_1_, ∇_1_, *Q*_2_, *N*_2_) ${\tilde{⊓}}_{\varepsilon }$ (Δ, ∇, *Q*_1_, *N*_1_).
3. (Δ, ∇, *Q*_1_, *N*_1_) ${\tilde{⊔}}_{ℜ}$ (Δ_1_, ∇_1_, *Q*_2_, *N*_2_) = (Δ_1_, ∇_1_, *Q*_2_, *N*_2_) ${\tilde{⊔}}_{ℜ}$ (Δ, ∇, *Q*_1_, *N*_1_).
4. (Δ, ∇, *Q*_1_, *N*_1_) ${\tilde{⊓}}_{ℜ}$ (Δ_1_, ∇_1_, *Q*_2_, *N*_2_) = (Δ_1_, ∇_1_, *Q*_2_, *N*_2_) ${\tilde{⊓}}_{ℜ}$ (Δ, ∇, *Q*_1_, *N*_1_).
5. (Δ, ∇, *Q*_1_, *N*_1_) ${\tilde{⊔}}_{\varepsilon }$ ((Δ_1_, ∇_1_, *Q*_2_, *N*_2_) ${\tilde{⊔}}_{\varepsilon }$ (Δ_2_, ∇_2_, *Q*_3_, *N*_3_)) = ((Δ, ∇, *Q*_1_, *N*_1_) ${\tilde{⊔}}_{\varepsilon }$ (Δ_1_, ∇_1_, *Q*_2_, *N*_2_)) ${\tilde{⊔}}_{\varepsilon }$ (Δ_2_, ∇_2_, *Q*_3_, *N*_3_).
6. (Δ, ∇, *Q*_1_, *N*_1_) ${\tilde{⊓}}_{\varepsilon }$ ((Δ_1_, ∇_1_, *Q*_2_, *N*_2_) ${\tilde{⊓}}_{\varepsilon }$ (Δ_2_, ∇_2_, *Q*_3_, *N*_3_)) = ((Δ, ∇, *Q*_1_, *N*_1_) ${\tilde{⊓}}_{\varepsilon }$ (Δ_1_, ∇_1_, *Q*_2_, *N*_2_)) ${\tilde{⊓}}_{\varepsilon }$ (Δ_2_, ∇_2_, *Q*_3_, *N*_3_).
7. (Δ, ∇, *Q*_1_, *N*_1_) ${\tilde{⊔}}_{ℜ}$ ((Δ_1_, ∇_1_, *Q*_2_, *N*_2_) ${\tilde{⊔}}_{ℜ}$ (Δ_2_, ∇_2_, *Q*_3_, *N*_3_)) = ((Δ, ∇, *Q*_1_, *N*_1_) ${\tilde{⊔}}_{ℜ}$ (Δ_1_, ∇_1_, *Q*_2_, *N*_2_)) ${\tilde{⊔}}_{ℜ}$ (Δ_2_, ∇_2_, *Q*_3_, *N*_3_).
8. (Δ, ∇, *Q*_1_, *N*_1_) ${\tilde{⊓}}_{ℜ}$ ((Δ_1_, ∇_1_, *Q*_2_, *N*_2_) ${\tilde{⊓}}_{ℜ}$ (Δ_2_, ∇_2_, *Q*_3_, *N*_3_)) = ((Δ, ∇, *Q*_1_, *N*_1_) ${\tilde{⊓}}_{ℜ}$ (Δ_1_, ∇_1_, *Q*_2_, *N*_2_)) ${\tilde{⊓}}_{ℜ}$ (Δ_2_, ∇_2_, *Q*_3_, *N*_3_).

*Proof*. Straightforward.

**Proposition 7**. *Let* (Δ, ∇, *Q*_1_, *N*) *and* (Δ_1_, ∇_1_, *Q*_2_, *N*) *be two N-BHS sets. Then*,

1. (Δ, ∇, *Q*_1_, *N*) ${\tilde{⊔}}_{\varepsilon }$ ((Δ, ∇, *Q*_1_, *N*) ${\tilde{⊓}}_{ℜ}$ (Δ_1_, ∇_1_, *Q*_2_, *N*)) = (Δ, ∇, *Q*_1_, *N*).
2. (Δ, ∇, *Q*_1_, *N*) ${\tilde{⊓}}_{\varepsilon }$ ((Δ, ∇, *Q*_1_, *N*) ${\tilde{⊔}}_{ℜ}$ (Δ_1_, ∇_1_, *Q*_2_, *N*)) = (Δ, ∇, *Q*_1_, *N*).
3. (Δ, ∇, *Q*_1_, *N*) ${\tilde{⊔}}_{ℜ}$ ((Δ, ∇, *Q*_1_, *N*) ${\tilde{⊓}}_{\varepsilon }$ (Δ_1_, ∇_1_, *Q*_2_, *N*)) = (Δ, ∇, *Q*_1_, *N*).
4. (Δ, ∇, *Q*_1_, *N*) ${\tilde{⊓}}_{ℜ}$ ((Δ, ∇, *Q*_1_, *N*) ${\tilde{⊔}}_{\varepsilon }$ (Δ_1_, ∇_1_, *Q*_2_, *N*)) = (Δ, ∇, *Q*_1_, *N*).

*Proof*. Straightforward.

**Proposition 8**. *Let* (Δ, ∇, *Q*_1_, *N*_1_), (Δ_1_, ∇_1_, *Q*_2_, *N*_2_), *and* (Δ_2_, ∇_2_, *Q*_3_, *N*_3_) *be three N-BHS sets. Then*,

1. (Δ, ∇, *Q*_1_, *N*_1_) ${\tilde{⊔}}_{\varepsilon }$ ((Δ_1_, ∇_1_, *Q*_2_, *N*_2_) ${\tilde{⊓}}_{ℜ}$ (Δ_2_, ∇_2_, *Q*_3_, *N*_3_)) = ((Δ, ∇, *Q*_1_, *N*_1_) ${\tilde{⊔}}_{\varepsilon }$ (Δ_1_, ∇_1_, *Q*_2_, *N*_2_)) ${\tilde{⊓}}_{ℜ}$ ((Δ, ∇, *Q*_1_, *N*_1_) ${\tilde{⊔}}_{\varepsilon }$ (Δ_2_, ∇_2_, *Q*_3_, *N*_3_)).
2. (Δ, ∇, *Q*_1_, *N*_1_) ${\tilde{⊓}}_{ℜ}$ ((Δ_1_, ∇_1_, *Q*_2_, *N*_2_) ${\tilde{⊔}}_{\varepsilon }$ (Δ_2_, ∇_2_, *Q*_3_, *N*_3_)) = ((Δ, ∇, *Q*_1_, *N*_1_) ${\tilde{⊓}}_{ℜ}$ (Δ_1_, ∇_1_, *Q*_2_, *N*_2_)) ${\tilde{⊔}}_{\varepsilon }$ ((Δ, ∇, *Q*_1_, *N*_1_) ${\tilde{⊓}}_{ℜ}$ (Δ_2_, ∇_2_, *Q*_3_, *N*_3_)).
3. (Δ, ∇, *Q*_1_, *N*_1_) ${\tilde{⊓}}_{\varepsilon }$ ((Δ_1_, ∇_1_, *Q*_2_, *N*_2_) ${\tilde{⊔}}_{ℜ}$ (Δ_2_, ∇_2_, *Q*_3_, *N*_3_)) = ((Δ, ∇, *Q*_1_, *N*_1_) ${\tilde{⊓}}_{\varepsilon }$ (Δ_1_, ∇_1_, *Q*_2_, *N*_2_)) ${\tilde{⊔}}_{ℜ}$ ((Δ, ∇, *Q*_1_, *N*_1_) ${\tilde{⊓}}_{\varepsilon }$ (Δ_2_, ∇_2_, *Q*_3_, *N*_3_)).
4. (Δ, ∇, *Q*_1_, *N*_1_) ${\tilde{⊔}}_{ℜ}$ ((Δ_1_, ∇_1_, *Q*_2_, *N*_2_) ${\tilde{⊓}}_{\varepsilon }$ (Δ_2_, ∇_2_, *Q*_3_, *N*_3_)) = ((Δ, ∇, *Q*_1_, *N*_1_) ${\tilde{⊔}}_{ℜ}$ (Δ_1_, ∇_1_, *Q*_2_, *N*_2_)) ${\tilde{⊓}}_{\varepsilon }$ ((Δ, ∇, *Q*_1_, *N*_1_) ${\tilde{⊔}}_{ℜ}$ (Δ_2_, ∇_2_, *Q*_3_, *N*_3_)).
5. (Δ, ∇, *Q*_1_, *N*_1_) ${\tilde{⊔}}_{ℜ}$ ((Δ_1_, ∇_1_, *Q*_2_, *N*_2_) ${\tilde{⊓}}_{ℜ}$ (Δ_2_, ∇_2_, *Q*_3_, *N*_3_)) = ((Δ, ∇, *Q*_1_, *N*_1_) ${\tilde{⊔}}_{ℜ}$ (Δ_1_, ∇_1_, *Q*_2_, *N*_2_)) ${\tilde{⊓}}_{ℜ}$ ((Δ, ∇, *Q*_1_, *N*_1_) ${\tilde{⊔}}_{ℜ}$ (Δ_2_, ∇_2_, *Q*_3_, *N*_3_)).
6. (Δ, ∇, *Q*_1_, *N*_1_) ${\tilde{⊓}}_{ℜ}$ ((Δ_1_, ∇_1_, *Q*_2_, *N*_2_) ${\tilde{⊔}}_{ℜ}$ (Δ_2_, ∇_2_, *Q*_3_, *N*_3_)) = ((Δ, ∇, *Q*_1_, *N*_1_) ${\tilde{⊓}}_{ℜ}$ (Δ_1_, ∇_1_, *Q*_2_, *N*_2_)) ${\tilde{⊔}}_{ℜ}$ ((Δ, ∇, *Q*_1_, *N*_1_) ${\tilde{⊓}}_{ℜ}$ (Δ_2_, ∇_2_, *Q*_3_, *N*_3_)).

*Proof*. (4) Suppose that ((Δ_1_, ∇_1_, *Q*_2_, *N*_2_) ${\tilde{⊓}}_{\varepsilon }$ (Δ_2_, ∇_2_, *Q*_3_, *N*_3_)) = (Δ_3_, ∇_3_, *Q*_2_ ∪ *Q*_3_, *max*(*N*_2_, *N*_3_)), then for all *q* ∈ *Q*_2_ ∪ *Q*_3_ and *ω* ∈ Ω:

$$\Delta_{3}\left(q\right)\left(\omega \right)=\{\begin{array}{cc} \Delta_{1}\left(q\right)\left(\omega \right) & \text{if}\;q\in Q_{2}\backslash Q_{3}\\ \Delta_{2}\left(q\right)\left(\omega \right) & \text{if}\;q\in Q_{3}\backslash Q_{2}\\ min\{\Delta_{1}\left(q\right)\left(\omega \right),\Delta_{2}\left(q\right)\left(\omega \right)\} & \text{if}\;q\in Q_{2}\cap Q_{3} \end{array}$$

and

$$\nabla_{3}\left(\neg q\right)\left(\omega \right)=\{\begin{array}{cc} \nabla_{1}\left(\neg q\right)\left(\omega \right) & \text{if}\;\neg q\in \neg Q_{2}\backslash \neg Q_{3}\\ \nabla_{2}\left(\neg q\right)\left(\omega \right) & \text{if}\;\neg q\in \neg Q_{3}\backslash \neg Q_{2}\\ max\{\nabla_{1}\left(\neg q\right)\left(\omega \right),\nabla_{2}\left(\neg q\right)\left(\omega \right)\} & \text{if}\;\neg q\in \neg Q_{2}\cap \neg Q_{3} \end{array}$$

Let (Δ, ∇, *Q*_1_, *N*_1_) ${\tilde{⊔}}_{ℜ}$ (Δ_3_, ∇_3_, *Q*_2_ ∪ *Q*_3_, *max*(*N*_2_, *N*_3_)) = (Δ_4_, ∇_4_, *Q*_1_ ∩ (*Q*_2_ ∪ *Q*_3_), *max*(*N*_1_, *max*(*N*_2_, *N*_3_))) = (Δ_3_, ∇_3_, *O* ∪ *P*, *max*(*N*_1_, *N*_2_, *N*_3_)) where *O* = *Q*_1_ ∩ *Q*_2_ and *P* = *Q*_1_ ∩ *Q*_3_, then for all *q* ∈ *O* ∪ *P* and *ω* ∈ Ω:

Δ_4_(*q*)(*ω*) = *max*{Δ(*q*)(*ω*), Δ_3_(*q*)(*ω*)} and ∇_4_(¬*q*)(*ω*) = *min*{∇(¬*q*)(*ω*), ∇_3_(¬*q*)(*ω*)}.

Hence,

$$\Delta_{4}\left(q\right)\left(\omega \right)=\{\begin{array}{cc} max\{\Delta \left(q\right)\left(\omega \right),\Delta_{1}\left(q\right)\left(\omega \right)\} & \text{if}\;q\in O\backslash P\\ max\{\Delta \left(q\right)\left(\omega \right),\Delta_{2}\left(q\right)\left(\omega \right)\} & \text{if}\;q\in P\backslash O\\ max\{\Delta \left(q\right)\left(\omega \right),min\left\{\Delta_{1}\left(q\right)\left(\omega \right),\Delta_{2}\left(q\right)\left(\omega \right)\right\}\} & \text{if}\;q\in O\cap P \end{array}$$

and

$$\nabla_{4}\left(\neg q\right)\left(\omega \right)=\{\begin{array}{cc} min\{\nabla \left(\neg q\right)\left(\omega \right),\nabla_{1}\left(\neg q\right)\left(\omega \right)\} & \text{if}\;\neg q\in \neg O\backslash \neg P\\ min\{\nabla \left(\neg q\right)\left(\omega \right),\nabla_{2}\left(\neg q\right)\left(\omega \right)\} & \text{if}\;\neg q\in \neg P\backslash \neg O\\ min\{\nabla \left(\neg q\right)\left(\omega \right),max\left\{\nabla_{1}\left(\neg q\right)\left(\omega \right),\nabla_{2}\left(\neg q\right)\left(\omega \right)\right\}\} & \text{if}\;\neg q\in \neg O\cap \neg P \end{array}$$

On the other hand, let (Δ, ∇, *Q*_1_, *N*_1_) ${\tilde{⊔}}_{ℜ}$ (Δ_1_, ∇_1_, *Q*_2_, *N*_2_) = (Δ_5_, ∇_5_, *Q*_1_ ∩ *Q*_2_, *max*(*N*_1_, *N*_2_)), then for all *q* ∈ *Q*_1_ ∩ *Q*_2_ and *ω* ∈ Ω:

Δ_5_(*q*)(*ω*) = *max*{Δ(*q*)(*ω*), Δ_1_(*q*)(*ω*)} and ∇_5_(¬*q*)(*ω*) = *min*{∇(¬*q*)(*ω*), ∇_1_(¬*q*)(*ω*)}.

Let (Δ, ∇, *Q*_1_, *N*_1_) ${\tilde{⊔}}_{ℜ}$ (Δ_2_, ∇_2_, *Q*_3_, *N*_3_) = (Δ_6_, ∇_6_, *Q*_1_ ∩ *Q*_3_, *max*(*N*_1_, *N*_3_)), then for all *q* ∈ *Q*_1_ ∩ *Q*_3_ and *ω* ∈ Ω:

Δ_6_(*q*)(*ω*) = *max*{Δ(*q*)(*ω*), Δ_2_(*q*)(*ω*)} and ∇_6_(¬*q*)(*ω*) = *min*{∇(¬*q*)(*ω*), ∇_2_(¬*q*)(*ω*)}.

Now, suppose that (Δ_5_, ∇_5_, *Q*_1_ ∩ *Q*_2_, *max*(*N*_1_, *N*_2_)) ${\tilde{⊓}}_{\varepsilon }$ (Δ_6_, ∇_6_, *Q*_1_ ∩ *Q*_3_, *max*(*N*_1_, *N*_3_)) = (Δ_7_, ∇_7_, *O* ∪ *P*), *max*(*N*_1_, *N*_2_, *N*_3_)) where *O* = *Q*_1_ ∩ *Q*_2_ and *P* = *Q*_1_ ∩ *Q*_3_, then for all *q* ∈ *O* ∪ *P* and *ω* ∈ Ω:

$$\Delta_{7}\left(q\right)\left(\omega \right)=\{\begin{array}{cc} \Delta_{5}\left(q\right)\left(\omega \right) & \text{if}\;q\in O\backslash P\\ \Delta_{6}\left(q\right)\left(\omega \right) & \text{if}\;q\in P\backslash O\\ min\{\Delta_{5}\left(q\right)\left(\omega \right),\Delta_{6}\left(q\right)\left(\omega \right)\} & \text{if}\;q\in O\cap P \end{array}$$

$$=\{\begin{array}{cc} max\{\Delta \left(q\right)\left(\omega \right),\Delta_{1}\left(q\right)\left(\omega \right)\} & \text{if}\;q\in O\backslash P\\ max\{\Delta \left(q\right)\left(\omega \right),\Delta_{2}\left(q\right)\left(\omega \right)\} & \text{if}\;q\in P\backslash O\\ min\{max\left\{\Delta \left(q\right)\left(\omega \right),\Delta_{1}\left(q\right)\left(\omega \right)\right\},max\left\{\Delta \left(q\right)\left(\omega \right),\Delta_{2}\left(q\right)\left(\omega \right)\right\}\} & \text{if}\;q\in O\cap P \end{array}$$

and

$$\nabla_{7}\left(\neg q\right)\left(\omega \right)=\{\begin{array}{cc} \nabla_{5}\left(\neg q\right)\left(\omega \right) & \text{if}\;\neg q\in \neg O\backslash \neg P\\ \nabla_{6}\left(\neg q\right)\left(\omega \right) & \text{if}\;\neg q\in \neg P\backslash \neg O\\ max\{\nabla_{5}\left(\neg q\right)\left(\omega \right),\nabla_{6}\left(\neg q\right)\left(\omega \right)\} & \text{if}\;\neg q\in \neg O\cap \neg P \end{array}$$

$$=\{\begin{array}{cc} min\{\nabla \left(\neg q\right)\left(\omega \right),\nabla_{1}\left(\neg q\right)\left(\omega \right)\} & \text{if}\;\neg q\in \neg O\backslash \neg P\\ min\{\nabla \left(\neg q\right)\left(\omega \right),\nabla_{2}\left(\neg q\right)\left(\omega \right)\} & \text{if}\;\neg q\in \neg P\backslash \neg O\\ max\{min\left\{\nabla \left(\neg q\right)\left(\omega \right),\nabla_{1}\left(\neg q\right)\left(\omega \right)\right\},min\left\{\nabla \left(\neg q\right)\left(\omega \right),\nabla_{2}\left(\neg q\right)\left(\omega \right)\right\}\} & \text{if}\;\neg q\in \neg O\cap \neg P \end{array}$$

Since (Δ_4_, ∇_4_, *O* ∪ *P*, *max*(*N*_1_, *N*_2_, *N*_3_)) and (Δ_7_, ∇_7_, *O* ∪ *P*, *max*(*N*_1_, *N*_2_, *N*_3_)) are equivalent for all *q* ∈ *O* ∪ *P* and *ω* ∈ Ω, the proof is concluded.

The other parts can be proven in a similar manner.

## 5 Enhancing decision-making through *N*-bipolar hypersoft sets

The decision-making approach described in this section extends the decision-making approach for soft sets, as discussed in the reference [5], while retaining all the relevant parameters. Algorithms 1 and 2 are employed to rank the alternatives based on their extended choice values and extended weight choice values, respectively. Moreover, Algorithm 3 illustrates the possibility of ranking the alternatives in the universe Ω using the information contained in (Δ, ∇, *Q*_1_, *N*) and a specified threshold *T*. In the following, a comprehensive explanation of the step-by-step process involved in these algorithms is provided.

**Algorithm 1**. Extended Choice Values.

1. **Input**:

 (a) Ω = {*ω*_*i*_, *i* = 1, 2, …, *m*} and *Q*_1_ = {*q*_*j*_, *j* = 1, 2, …, *n*}.

 (b) The *N*-BHS set (Δ, ∇, *Q*_1_, *N*) with *R* = {0, 1, …, *N* − 1} where *N* ∈ {2, 3, …} such that for all *ω*_*i*_ ∈ Ω, *q*_*j*_ ∈ *Q*_1_ there exists $r_{ij}^{+}$, $r_{ij}^{-}$ ∈ *R*.

2. For each *ω*_*i*_, compute $\sigma_{i}=\sigma_{i}^{+}$ − $\sigma_{i}^{-}$ where $\sigma_{i}^{+}=\sum_{j=1}^{n}r_{ij}^{+}$ and $\sigma_{i}^{-}=\sum_{j=1}^{n}r_{ij}^{-}$.

3. Find *k* such that *σ*_*k*_ = *max σ*_*i*_.

**Output**: The output is any *ω*_*k*_ that satisfies the condition in step 3.

By applying Algorithm 1 to Example 1 in Table 12, the conclusion can be made that the candidate *ω*_1_ is selected as the result. Therefore, the ranking decision based on the algorithm is *ω*_1_ > *ω*_5_ > *ω*_2_ > *ω*_3_ > *ω*_4_.

**Table 12:** The extended choice values in Example 1.

(Δ, ∇, *Q*_1_, 6)	*q*_1_	*q*_2_	*q*_3_	*q*_4_	*q*_5_	*q*_6_	*σ*_*i*_
*ω*_1_	(4,1)	(5,0)	(3,2)	(5,0)	(4,1)	(3,2)	12
*ω*_2_	(2,1)	(4,1)	(1,4)	(3,1)	(4,1)	(5,0)	3
*ω*_3_	(0,0)	(1,1)	(3,1)	(0,2)	(4,0)	(3,1)	1
*ω*_4_	(0,5)	(3,2)	(2,2)	(5,0)	(5,0)	(4,1)	-1
*ω*_5_	(4,0)	(5,0)	(0,1)	(1,3)	(4,1)	(4,0)	8

**Algorithm 2**. Extended Weight Choice Values.

1. **Input**:

 (a) Ω = {*ω*_*i*_, *i* = 1, 2, …, *m*}, *Q*_1_ = {*q*_*j*_, *j* = 1, 2, …, *n*}, and a weight *W*_*j*_ for each parameter.

 (b) The *N*-BHS set (Δ, ∇, *Q*_1_, *N*) with *R* = {0, 1, …, *N* − 1} where *N* ∈ {2, 3, …} such that for all *ω*_*i*_ ∈ Ω, *q*_*j*_ ∈ *Q*_1_ there exists $r_{ij}^{+}$, $r_{ij}^{-}$ ∈ *R*.

2. For each *ω*_*i*_, compute $\sigma_{i}^{W}=\sigma_{i}^{+W}$ − $\sigma_{i}^{-W}$ where $\sigma_{i}^{+W}=\sum_{j=1}^{n}r_{ij}^{+}$ *W*_*j*_ and $\sigma_{i}^{-W}=\sum_{j=1}^{n}r_{ij}^{-}$ (1−*W*_*j*_).

3. Find *k* such that $\sigma_{k}^{W}=max\;\sigma_{i}^{W}$.

**Output**: The output is any *ω*_*k*_ that satisfies the condition in step 3.

**Example 5**. *Given the weights W*_1_ = 0.8, *W*_2_ = 0.6, *W*_3_ = 0.6, *W*_4_ = 0.5, *W*_5_ = 0.7, *and W*_6_ = 0.6 *assigned to the skills q*_*j*_ *in Example 1, the analysis from* Table 13 *indicates that the candidate ω*_1_ *is chosen, and the ranking decision is ω*_1_ > *ω*_5_ > *ω*_2_ > *ω*_4_ > *ω*_3_.

**Table 13:** The extended weight choice values in Example 5.

(Δ, ∇, *Q*_1_, 6)	*q*_1_	*q*_2_	*q*_3_	*q*_4_	*q*_5_	*q*_6_	$\sigma_{i}^{W}$
*ω*_1_	(3.2,0.2)	(3,0)	(1.8,0.8)	(2.5,0)	(2.8,0.3)	(1.8,0.8)	13
*ω*_2_	(1.6,0.2)	(2.4,0.4))	(0.6,1.6)	(1.5,0.5)	(2.8,0.3)	(3,0)	8.9
*ω*_3_	(0,0)	(0.6,0.4)	(1.8,0.4)	(0,1)	(2.8,0)	(1.8,0.4)	5.7
*ω*_4_	(0,1)	(1.8,0.8)	(1.2,0.8)	(2.5,0)	(3.5,0)	(2.4,0.4)	8.4
*ω*_5_	(3.2,0)	(3,0)	(0,0.4)	(0.5,1.5)	(2.8,0.3)	(2.4,0)	9.7

**Remark 5**. *It can be easily verified that if two N-BHS sets* (Δ, ∇, *Q*_1_, *N*) *and* (Δ_1_, ∇_1_, *Q*_2_, *N*) *are equivalent under normalization, then the ranking of the alternatives in* Ω *based on their extended choice values will remain unchanged for both N-BHS sets*.

**Example 6**. *By referring to* Table 14, *which presents the 4-choice values* (Δ^4^, ∇^4^, *Q*_1_) *of* (Δ, ∇, *Q*_1_, 6) *in Example 1, it can be inferred that the candidate ω*_5_ *is chosen and the ranking decision is ω*_5_ > *ω*_1_ = *ω*_2_ > *ω*_3_ = *ω*_4_.

**Table 14:** The 4-choice values in Example 6.

(Δ^4^, ∇^4^, *Q*_1_)	*q*_1_	*q*_2_	*q*_3_	*q*_4_	*q*_5_	*q*_6_	$\sigma_{i}^{4}$
*ω*_1_	(1,0)	(1,0)	(0,1)	(1,0)	(1,0)	(0,1)	2
*ω*_2_	(0,0)	(1,0)	(0,1)	(0,0)	(1,0)	(1,0)	2
*ω*_3_	(0,0)	(0,0)	(0,0)	(0,1)	(1,0)	(0,0)	0
*ω*_4_	(0,1)	(0,1)	(0,1)	(1,0)	(1,0)	(1,0)	0
*ω*_5_	(1,0)	(1,0)	(0,0)	(0,1)	(1,0)	(1,0)	3

**Algorithm 3**. *T*-Choice Values.

1. **Input**:

 (a) Ω = {*ω*_*i*_, *i* = 1, 2, …, *m*} and *Q*_1_ = {*q*_*j*_, *j* = 1, 2, …, *n*}.

 (b) The *N*-BHS set (Δ, ∇, *Q*_1_, *N*) with *R* = {0, 1, …, *N* − 1} where *N* ∈ {2, 3, …} such that for all *ω*_*i*_ ∈ Ω, *q*_*j*_ ∈ *Q*_1_ there exists $r_{ij}^{+}$, $r_{ij}^{-}\in R$.

 (c) The threshold *T*.

2. Compute $r_{ij}^{+T}$ and $r_{ij}^{-T}$ where

$$r_{ij}^{+T}=\{\begin{array}{cc} 1, & \text{if}\;r_{ij}^{+}\geq T\\ 0, & \text{otherwise} \end{array}$$

 and

$$r_{ij}^{-T}=\{\begin{array}{cc} 1, & \text{if}\;r_{ij}^{-}\geq \text{N}-T\\ 0, & \text{otherwise} \end{array}$$

3. For each *ω*_*i*_, compute $\sigma_{i}^{\tilde{T}}=\sigma_{i}^{+T}$ − $\sigma_{i}^{-T}$ where $\sigma_{i}^{+T}=\sum_{j=1}^{n}r_{ij}^{+T}$ and $\sigma_{i}^{-T}=\sum_{j=1}^{n}r_{ij}^{-T}$.

4. Find *k* such that $\sigma_{k}^{\tilde{T}}=max\;\sigma_{i}^{\tilde{T}}$.

**Output**: The output is any *ω*_*k*_ that satisfies the condition in step 4.

## 6 Comparative analysis

In this section, Examples 1, 5, and 6 are employed to assess and compare the results generated by the algorithms. By examining the outcomes produced by the three algorithms (as presented in Table 15), significant variations are observed. This stark contrast in results serves as compelling evidence, highlighting the adaptability and versatility embedded within the decision-making algorithms.

**Table 15:** Comparison of Algorithms 1, 2 and 3 using Examples 1, 5, and 6.

Algorithm No.	Algorithm Name	Decision Ranking
Algorithm 1	Extended Choice Values	*ω*_1_ > *ω*_5_ > *ω*_2_ > *ω*_3_ > *ω*_4_
Algorithm 2	Extended Weight Choice Values	*ω*_1_ > *ω*_5_ > *ω*_2_ > *ω*_4_ > *ω*_3_
Algorithm 3	*T*-Choice Values	*ω*_5_ > *ω*_1_ = *ω*_2_ > *ω*_3_ = *ω*_4_

Now, we present a comparative analysis of the proposed *N*-BHS set model with relevant existing models, considering key evaluation factors such as SF (single-argument approximate function), MF (multi-argument approximate function), BS (bipolarity setting), and NBD (non-binary and discrete data). The objective is to highlight the versatility and effectiveness of the *N*-BHS set model in addressing these crucial features compared to other models in the field.

Table 16 provides a comprehensive comparison, showcasing the applicability or limitations of each model for the identified evaluation factors. The presence of a checkmark (✓) indicates that the model successfully incorporates the respective feature, while the symbol (×) signifies its absence or limited implementation.

**Table 16:** Comparison of the *N*-BHS set model with relevant existing models.

Authors	Models	SF	MF	BS	NBD
Molodtsov [4]	Soft sets	✓	×	×	×
Fatimah et al. [17]	*N*-Soft sets	✓	×	×	✓
Shabir et al. [14]	Bipolar soft sets	✓	×	✓	×
Shabir et al. [18]	*N*-Bipolar soft sets	✓	×	✓	✓
Smarandache [32]	HS sets	✓	✓	×	×
Musa et al. [40]	*N*-HS sets	✓	✓	×	✓
Musa et al. [41]	BHS sets	✓	✓	✓	×
Proposed model	*N*-BHS sets	✓	✓	✓	✓

The analysis clearly demonstrates that the *N*-BHS set model effectively addresses all the evaluated features, making it a comprehensive and versatile approach. On the other hand, the comparative models exhibit limitations in one or more of these aspects, highlighting the unique strengths and advancements offered by the *N*-BHS set model.

By conducting this comparative analysis, we establish the superiority of the proposed *N*-BHS set model in terms of its ability to incorporate SF, MF, BS, and NBD, thereby distinguishing it from existing models in the field. This analysis reinforces the significance and relevance of our research, providing valuable insights for further advancement and adoption of the *N*-BHS set model in practical applications.

## 7 Concluding remarks

Motivated by identified limitations in existing literature, this study introduces *N*-BHS sets as a novel framework designed to address challenges in managing evaluations involving both binary and non-binary data. Utilizing two key tools—presenting a nuanced parameterized representation of the universe with finite granularity in perceiving attributes and strategically partitioning attributes into distinct subattribute values using disjoint sets—the *N*-BHS sets emerge as a versatile solution for effectively handling uncertainty-related problems. In addition to advancing the theoretical foundations of the proposed model, this study presents practical methodologies, encompassing various algebraic definitions, set-theoretic operations, and decision-making approaches specific to *N*-BHS sets. The conclusion provides a comprehensive comparison with relevant existing models, highlighting the distinct advancements facilitated by the introduction of *N*-BHS sets.

As a promising avenue for further investigation, we recommend extending the proposed N-BHS set model to incorporate fuzzy N-BHS sets. The integration of fuzzy logic principles into the existing N-BHS set framework holds the potential to enhance the model’s ability to handle uncertainty and imprecision within the evaluation framework. This extension enables the representation of evaluations with degrees of membership, providing a more comprehensive and nuanced approach.

Furthermore, exploring extensions such as fuzzy N-BHS expert sets, spherical fuzzy N-BHS expert sets, and Pythagorean fuzzy N-BHS expert sets can contribute to the versatility and applicability of the N-BHS set model. Additionally, we encourage the exploration of topological and algebraic structures using the proposed model, opening avenues for a deeper understanding of the underlying mathematical foundations.

These proposed extensions and investigations are anticipated to contribute to a more robust and flexible N-BHS set model, with potential applications in diverse domains. We look forward to revisiting these matters in future research endeavors.
